# Decision making tools for managing waiting times and treatment rates in elective surgery

**DOI:** 10.1186/s12913-019-4199-6

**Published:** 2019-06-11

**Authors:** Daniel Adrian Lungu, Tommaso Grillo Ruggieri, Sabina Nuti

**Affiliations:** Healthcare Management Laboratory, Institute of Management and Department EMbeDS, Scuola Superiore Sant’Anna, Piazza Martiri della Libertà, 33, Pisa, Italy

**Keywords:** Elective surgery, Waiting times, Use rates, Unwarranted variation, Decision making

## Abstract

**Background:**

Waiting times for elective treatments, including elective surgery, are a source of public concern and therefore are on policy makers’ agenda. The long waiting times have often been tackled through the allocation of additional resources, in an attempt to reduce them, but results are not straightforward. At the same time, researchers have reported wide geographical variations in the provision of elective care not driven by patient needs or preferences but by other factors.

The paper analyses the relationship between waiting times and treatment rates for nine high-volume elective surgical procedures in order to support decision making regarding the availability of these services for the citizens. Using the framework already proposed for the diagnostic services, we identify different patterns that can be followed to align the supply with patient needs in the Italian context.

**Methods:**

After measuring the waiting times and the treatment rates for nine procedures in the 34 districts in Tuscany, we performed correlation analyses. Then, we plotted the results in a matrix cross-checking waiting times and rates. By doing so, we identified four different contexts that require a second step analysis to tackle unwarranted geographical variations and ensure timely care to patients. Finally, for each district and elective surgical procedure, we measured the economic impact of the different treatment rates in order to evaluate whether there are any supply criticalities and eventually some room for maneuver. We also included active and passive mobility of patients.

**Results:**

The results show a high degree of variation both in treatment rates and waiting times, especially for the orthopaedic procedures: knee replacement, knee arthroscopy and hip replacement. The analysis performed for the nine interventions shows that the 34 districts are in varying positions in the waiting time-treatment rate matrix, suggesting that there is no straightforward relationship between rates and waiting times. Each combination in the matrix may have different determinants that require healthcare managers to adopt diversified strategies. The decision making process needs to be supported by a two-level analysis: the first one to put in place the matrix that cross-checks waiting times and treatment rates, the second one to analyse the characteristics of each quadrant and the improvement actions that can be proposed.

**Conclusions:**

In Italy, waiting times in elective surgical services are a main policy issue with a relevant geographical variation. Our analysis reveals that this variation is due to multiple elements. In order to avoid simplistic approaches that do not solve the problem but often lead to increased expenditure, policy makers and healthcare managers should follow a two-step strategy firstly identifying the type of context and secondly analysing the impact of elements such as resource productivity, resource availability, patients’ preferences and care appropriateness. Only in some cases it is required to increase the service supply.

**Electronic supplementary material:**

The online version of this article (10.1186/s12913-019-4199-6) contains supplementary material, which is available to authorized users.

## Background

The demand for elective surgical (ES) services has increased significantly above all due to the aging population, technological innovation, and the growing levels of patient expectations and trust in the favorable outcomes of surgical treatments. There have been significant increases in the volumes of cardiovascular, orthopaedic and vascular surgical procedures [1–3].

In ES delivery although, healthcare policy makers and managers have difficulties balancing the supply and demand. They struggle with widespread long waiting lists which are a source of public interest as they lead to dissatisfaction among citizens and raise equity concerns.

This is particularly relevant for ES treatments, since they are not provided in urgency conditions but are chosen by patients in agreement with their physicians in order to improve their quality of life. In fact, long waiting times for these services can drive patients to opt for private sector services or to renounce to treatment [4–9].

In Italy, according to the “Unmet health care needs statistics” report, 29.9% of population aged 15 or over in need for health care reported to have renounced to treatment due to long waiting lists (Eurostat, data extracted in January 2018). Thus, waiting times are taken very seriously by policy makers who are continuously seeking effective solutions, even though they focused mainly on the short-term.

These solutions to reduce waiting times have often entailed strengthening the supply side by increasing the resource availability (operating room hours, personnel and beds), by working on providers’ productivity or by paying extra money to the health professionals to increase production [4, 5]. Nevertheless, these solutions in the long term have failed to radically shorten waiting times at a system level [10, 11].

Several studies have highlighted that the healthcare system differs sharply from other industrial sectors, as the healthcare supply often drives the demand, and not the reverse [12–14]. In this sense, the question arises as to whether reducing ES waiting times requires interventions on the supply side only or whether there are other determinants that should be tackled. Hence, what analyses should be performed to identify the right actions to reduce waiting times and answer to patients’ needs? In our study we provide policy makers and managers with two-level tools to support decision making and prioritise the identification of actions aimed at shortening waiting times and reducing geographical unwarranted variations.

### Supply and demand for ES delivery

Beveridge healthcare systems pursuing universal coverage are supposed to provide an appropriate number of services to balance patient needs and simultaneously guarantee quality of care, timely interventions, and to ensure equity of access to all citizens.

Equity can be distinguished between its vertical and horizontal forms [15]. To achieve vertical equity, the healthcare system should recognise different health needs linked to specific factors such as the socio-economical characteristics (e.g. poverty) of populations or areas and allocate different amounts of resources in order to balance the different starting points with respect to other population groups and reduce inequalities.

To achieve horizontal equity, patients with the same healthcare needs should be able to count on the same care services. Hence, in horizontally unequal systems, for the same level of patient need, there are differences in the resource allocation and/or the quality of care. When this occurs between geographical areas, the so-called “postcode lottery” healthcare arises [16].

Evidence of horizontal inequity is given by unwarranted geographical variations in access rates (e.g. the hospital admission rates for specific treatments), which have been extensively reported in different contexts and for a wide-ranging list of different healthcare services [17–19].

A large extent of variation has been observed for the treatment rates of many elective surgical procedures, including hip and knee replacements [20] and laparoscopic colectomy [21]. Significant geographical variations have also been revealed for the outcomes of general and vascular surgery [22] and hip and knee arthroscopy [23].

Elective surgery includes both preference-sensitive and supply-sensitive elements and is thus connected to the potential risk of inappropriate healthcare delivery (under or over-treatment) as also suggested by the reported geographical variations. For instance, in some countries, 20 to 40% of joint arthroplasties were inappropriate according to the national evidence-based criteria [24, 25].

Scholars have widely discussed the different causes, categories and sources of these variations, which can be summarised through Wennberg’s taxonomy [18] integrated by Nuti et al. [26]:

1. Variation due to differences in providing effective clinically proven services to all patients (e.g. operations within 48 h of femur fractures for patients aged over 65), which is unwarranted since it highlights the healthcare system’s failure in ensuring effective care.
2. Variation in preference-sensitive services (e.g. elective surgical care for joint replacements), which is beneficial when it reflects patients’ different preferences rather than the physicians’ discretionary choices;
3. Variation in supply-sensitive services, where the treatment rates depend on the available resources in the different geographical areas (e.g. number of operation theatres, number of hospital beds, technologies for diagnostic imaging services), leading to unwarranted over or under-treatment.
4. Variation in care pathways that refers to services whose variation is due to a lack of effective care caused by the absence of integration through the entire care pathway.

Since ES services are driven by both patient needs and preferences as well as by supply-side factors, the search for solutions to shorten waiting times should also analyse unwarranted geographical variations, in order to detect the potential relationship between waiting lists and appropriateness [27].

Cross-checking the analysis of variations in treatment rates and waiting times across geographical areas could help to identify different scenarios and to propose consistent interventions based on the interrelation between these two phenomena and other factors.

This paper explores the relationship between waiting times and treatment rates for elective surgical services by jointly analysing their variations in Tuscany (central Italy), following the methodology already applied in the field of diagnostic services by Nuti and Vainieri [28].

Our objective is to provide policy makers with tools that go beyond the individual provider boundaries and that support the research of solutions aimed at shortening waiting times and driving resource (re) allocation accordingly.

## Methods

Our study focused on Tuscany and considered waiting times and treatment rates of nine elective (i.e. planned and not emergency cases) surgical procedures: knee replacement, hip replacement, percutaneous coronary angioplasty, hysterectomy, cholecystectomy, colectomy, transurethral prostatectomy, laparoscopic cholecystectomy, and knee arthroscopy. These procedures were chosen based on the volume ranking. A more detailed overview of the elective surgery provision in Tuscany is available in the Additional file 2.

We used administrative data to compute and then cross-check the two indicators (i.e. waiting times and treatment rates) considering the 2016 data at the district level.

We excluded patients that had been diagnosed with malignant tumors in order to include in our analysis only those treatments that do not require an intervention within a specific short time frame. In addition, ES procedures for cancer care highlight unquestionable patient needs, whose geographical variation does not necessarily reflect inappropriateness and merit a more in-depth analysis.

The treatment rates were computed as the number of procedures delivered for the inhabitants of a district divided by the number of inhabitants of that area and then multiplied by 100,000. The average waiting times for each district were computed as the sum of the number of days waited by each Tuscan patient from when the operation had been scheduled to the date of the planned hospital admission, divided by the number of Tuscan patients of each district. Both indicators consider the ES services provided for Tuscan citizens regardless whether they received the service in Tuscany or in other Italian regions.

With this data, we explored the relationship between waiting times and the treatment rates with three different strategies.

Our first step was to run the Pearson correlation test for each of the nine procedures using data at the district level (*n* = 34).

**Fig. 1:**
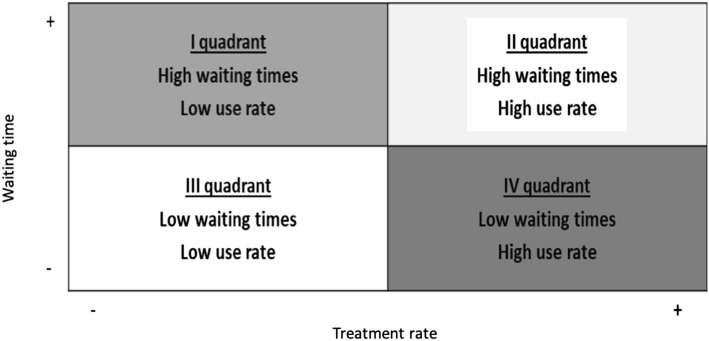
Logical framework for dealing with long waiting times and their relationship with treatment rates

In the second step, we used a graphical approach to visually explore the relationship between the two indicators, by plotting the performance of each district into a waiting times-treatment rates matrix for each of the nine procedures. Then, by using the framework proposed by Nuti and Vainieri (2012), we traced the median lines in order to obtain four quadrants (Fig. 1). We chose the matrix method because it provides a clear picture of the overall performance of a district. Even though a clinical standard does not exist for the treatment rates of these nine elective surgical services [29–32], the regional administration aims at reducing the unwarranted geographical variation to pursue equity for the citizens [33]. Plotting the organisations all together on the same matrix allowed us to obtain an intuitive estimation of the extent of geographical variation, with regard to both waiting times and treatment rates.

By cross-checking the treatment rates and the waiting times registered for the 34 districts we identified four different scenarios that require different strategies in order to reduce waiting times and inappropriateness. A limitation of the method is that reduction of waiting times implies that demand remains constant over time. Between 2014 and 2016 the demand for the nine elective interventions remained relatively constant, but policy makers should take into account that it may vary on the basis of several factors.

Furthermore, the matrix can be used as an identification model of the determinants of poor performance within each quadrant and help managers and policy makers to determine priorities when searching for solutions. Although the matrix can suggest priorities when looking for solutions, it does not provide conclusive evidence on where problems lie and therefore districts should use it as a starting point for more in depth analyses. By employing additional indicators of productive capacity and productivity together with the matrix, the regional administration would be able to spot the different determinants of poor performance and act accordingly.

The districts belonging to the first quadrant register long waiting times and relatively low treatment rates. The performance of these districts appears to be critical since their inhabitants face a two-fold problem: low treatment rates for the surgical procedure that could be symptom of under-treatment, but the ones who are operated also have to wait longer than other citizens.

Districts in the second quadrant face still long waiting times but manage to deliver high rates of ES procedures. Despite the potential risk of overuse highlighted by the high treatment rates, the long waiting times could also underline a potential efficiency problem in the use of resources.

Districts in the third quadrant may suffer from an underuse problem. In fact, waiting times are short but the treatment rates are significantly below the regional median.

Districts positioned in the fourth quadrant are characterised by high rates of ES procedures delivered and by short waiting times. Although patients benefit from prompt interventions, the high treatment rates raise appropriateness concerns and could also suggest overstaffing and oversupply.

In order to assess if in each district there was a homogeneous situation for all the ES procedures delivered, in the third step we built a summary table that shows the position of each district in the quintile ranking, considering the treatment rates and the average waiting times separately.

For each procedure, we divided the districts’ performance into quintiles and assigned a colour ranging from light blue (first quintile) to dark blue (fifth quintile). The summary table composed by the quintiles of the 34 districts (rows) and the nine procedures (columns) enables us to:

1. summarise the available information used in the matrixes (vertical reading);
2. prioritise interventions, by focusing on the extreme quintiles instead of the median value as a benchmark;
3. assess whether a district has a structural problem in terms of treatment rates and/or waiting times (horizontal reading). For instance, a district may register mainly high (or low) treatment rates for all the nine procedures: in this case, it may have organisational issues in both waiting times and appropriateness. If, on the other hand, a district registers both high and low values simultaneously, it may have problems in terms of the internal resource allocation among the different procedures (e.g. number of operating room hours).

The final step consisted, for each procedure, into the assessment of the value of the procedures that could be reallocated if the extent of geographical variation would be lesser.

The aim of this analysis was not to comprehensively assess the potential opportunities for resource reallocation in the elective surgery pathway, but to identify areas where a reduction of geographical variation could generate opportunities for resource reallocation.

We ran the simulation twice, following the previous work conducted by Nuti et al., in order to quantify the number of procedures that could be reduced if districts had achieved the regional average performance or the best performance in terms of treatment rates [34]. For each district, the number of procedures above the average or above the best performer was computed by multiplying the rate difference by the number of inhabitants. Finally, we computed the value of these procedures by multiplying their number by the DRG tariff.

## Results

The first step of the study consisted in investigating the relationship between waiting times and treatment rates for the nine ES procedures through the Pearson correlation test (Table 1).

**Table 1:**
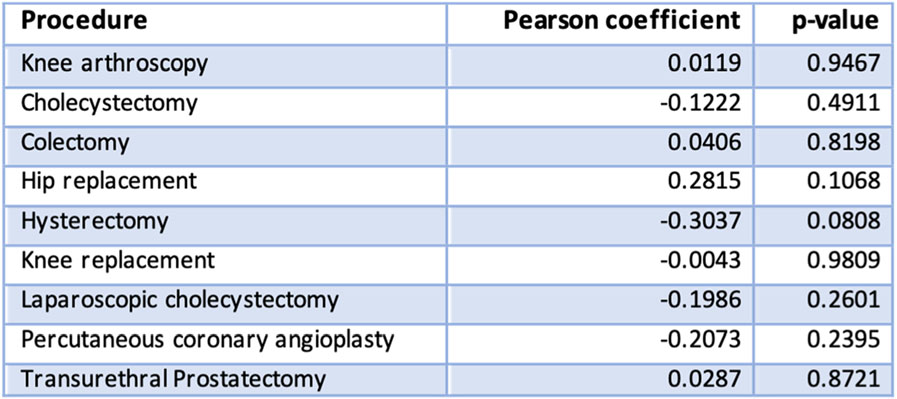
Waiting times – treatment rates correlation coefficients

The results show that the correlation coefficients between waiting times and the treatment rates are not significantly different from 0 since the *p*-value is consistently greater than 0.05. Thus, there appears to be no straightforward relationship between the two variables.

**Fig. 2:**
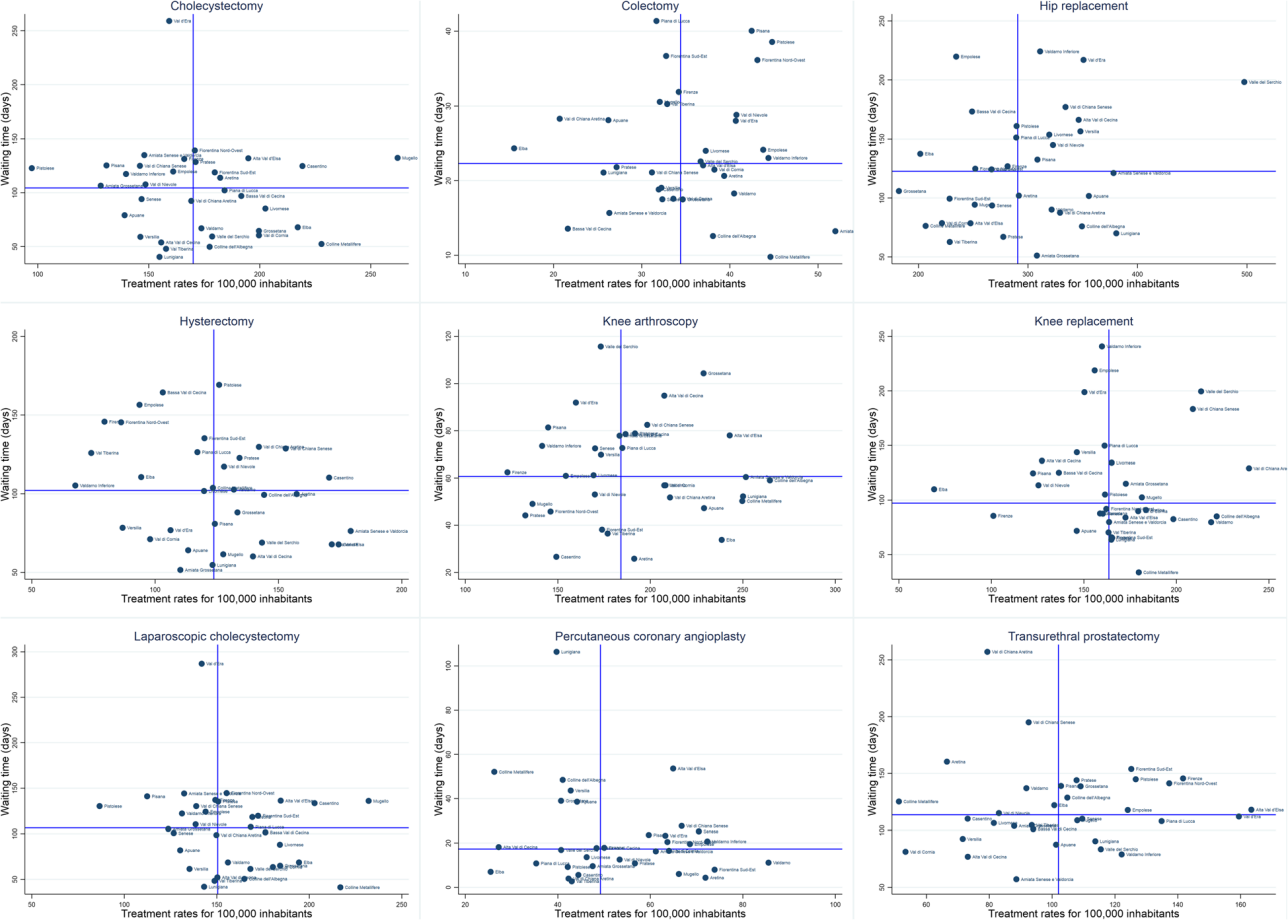
Waiting times – use rates matrixes for the nine elective surgical procedures

That given, the second step consisted into mapping the performance of the districts using the waiting times – treatment rates matrix presented in the Methods section. The results are summarised in Fig. 2, while a more detailed version is available in the Additional file 1.

For each procedure, there is considerable variation among the 34 districts in terms of both waits and rates. For example, hip and knee replacements present a 4/5-fold variation in waiting times and a 2/3-fold variation in treatment rates.

The results presented so far indicate that there is a great degree of heterogeneity among the Tuscan districts with regard to the waiting times and the treatment rates for ES services.

The third step of our analysis consisted into building the summary table (see the example in Table 2). The quintile approach is useful because it enables extreme values to be identified and it focuses on the most critical areas.

**Table 2:**
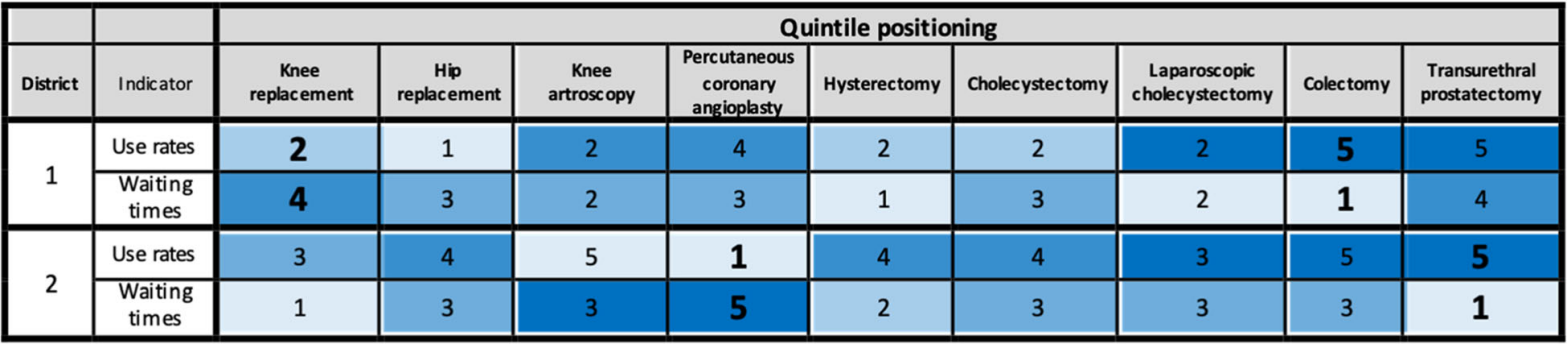
Example of the summary table of the 34 Tuscan districts in terms of treatment rates and waiting times

The two examples in Table 2 provide insights into how to use the information in the summary table to support ES delivery planning. Inhabitants of district No. 1 receive a low treatment rate (second quintile) and face a relatively long waiting time (fourth quintile) for knee replacements. Conversely, they experience a short waiting time (first quintile) and a relatively high rate (fifth quintile) for colectomy. A similar line of reasoning can be applied to the district No. 2 for the percutaneous coronary angioplasty and the transurethral prostatectomy procedures. The possible strategies that policy makers and managers can adopt are given in the next section.

The fourth and final step consisted in a simulation aimed at estimating the value of the procedures that could be avoided in case that geographical variation would be reduced. An example of the results are displayed in Table 3. The table shows the results for the hip replacement procedure, but the same analysis was performed also for the remaining eight elective surgical procedures.

**Table 3:**
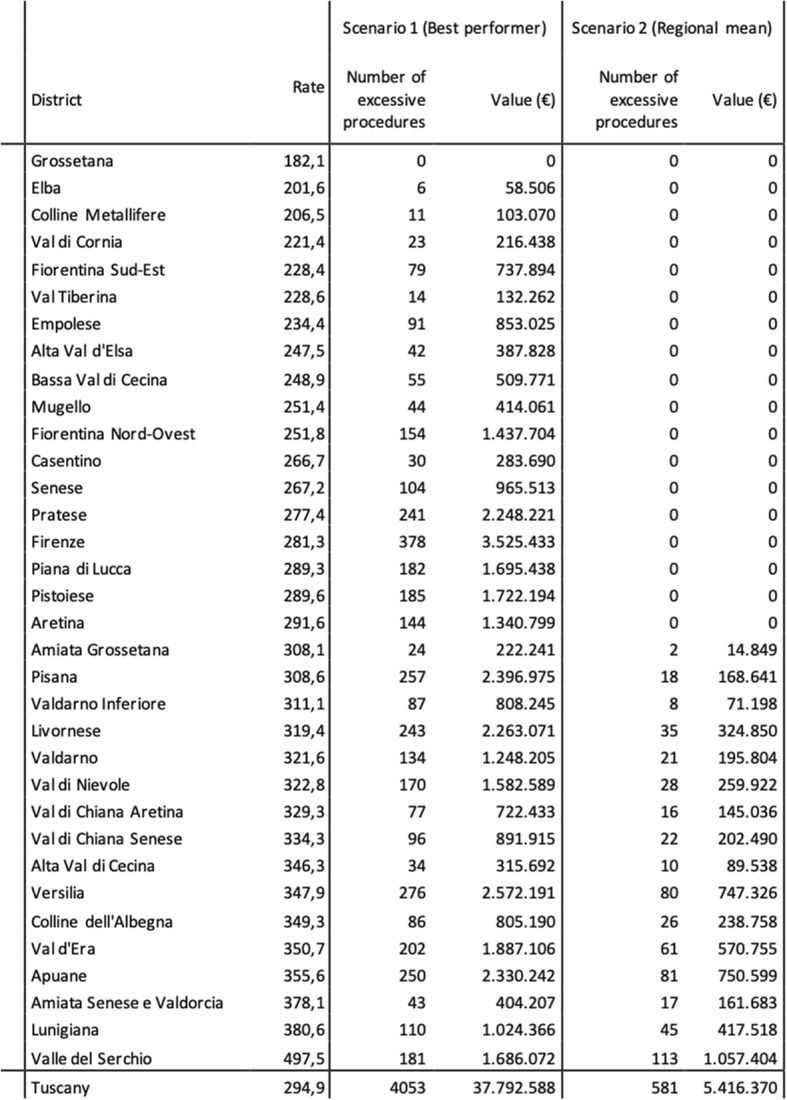
Estimation of the number and value of avoidable procedures, by district, if their performance would equal the best performance or the regional mean performance

The simulation consists of two scenarios: the first one hypothesizes that all districts reach the same rate as the best performer, while the second one estimates that districts that are above the mean regional treatment rate align to this latter performance.

The results of the first scenario display a significant degree of variation between the districts and therefore estimate that the value of procedures that could be reduced at a regional level is equal to € 37,792,588 per year, corresponding to a total of 4053 procedures.

The second scenario assumes that districts with greater treatment rates than the regional mean performance would decrease their rate in order to equal the regional mean rate. In this case, the number of hip replacement procedures that could be reduced is equal to 581, corresponding to a value of € 5,416,370.

Waiting times for elective surgery are a major health policy concern since citizens have to postpone treatment for several months. Beside the general dissatisfaction, the negative consequences include the potential worsening of health status and the postponed health benefits [35].

## Discussion

The absence of a price rationing mechanism to balance the supply and demand has highlighted that waiting times act as a form of non-price rationing that brings together the demand and the supply of health care [36, 37]. Theoretically, long waiting times might induce some patients to seek care from other providers and encourage hospitals to increase efficiency and deliver more care. The extent to which demand and supply are sensitive to waiting times is crucial from a policy perspective. In fact, the efficacy of strategic plans that aim at shortening waiting times depends on the capacity to identify the main determinants and the coherence of the strategy. In Tuscany, we found that the demand for elective surgery is not related to waiting times, confirming the results reported by previous studies.

Based on the data for nine elective surgical procedures performed in 34 health districts in Tuscany, we introduced two tools that show four different scenarios where supply-side interventions may have different impacts. Decisions on the reduction of waiting times should also consider the geographical variation in the treatment rates. Without cross-checking waiting times and treatment rates, supply-side interventions may reinforce the extent of geographical variation and not solve the problem of waiting lists. A detailed interpretation of the findings is available in the Additional file 2.

Beside the specific strategies suggested for each of the four quadrants, there are two elements that we consider essential for their success: physicians’ engagement and the patients’ preferences.

Physicians play a central role in the provision of ES services and influence the volume of procedures delivered by their prescribing behaviour; therefore, beside the policy makers, the tools to tackle unwarranted variation presented in this study are addressed also to clinicians [38]. The public disclosure of data, the discussion of results and the sharing of clinical guidelines among them could have a significant impact on reducing the inappropriateness of ES interventions.

Potential differences in treatment rates, with all other variables constant, might also reflect different approaches and preferences regarding ES procedures. As also suggested by a recent study trying to investigate unwarranted variation in ventilation tube insertions for otitis media with effusion in children in England, the identification of mechanisms such as Patient Reported Outcome Measures (PROMs) and Patient Reported Experience Measures (PREMs) could be beneficial to determine patients’ preferences and understand whether variations in care are justified by patient choices [39]. Although these mechanisms are not yet widespread, they could be helpful to take into account the patients’ perspective and obtain desirable variations.

The main limitations of this study consist in the fact that it only takes into account nine elective surgical procedures. However, we believe that the study can be easily replicated by including additional procedures or by considering other Italian or international areas. We do not think that the focus on Tuscany is a limitation since the performance of the 34 health districts is assessed by benchmarking their results. We chose to use the median value to provide an example of the methodology used to estimate the degree of geographical variation. Even when a clinical standard does not exist, as for the treatment rates of the nine surgical procedures analysed, the reduction of geographical unwarranted variation remains one of the equity goals that policy makers must pursue [33]. Moreover, the study does not address the heterogeneity in preference-sensitivity across the nine procedures. Indeed we suggest districts to tackle geographical unwarranted variation procedure by procedure, and by taking into account the patient perspective. The paper does not estimate the unmet needs of the nine procedures that could lead to part of the differences observed in treatment rates: although Tuscany is considered to be a homogeneous area from the socio-demographic point of view, some variations in prevalence of need might exist among the 34 districts.

## Conclusions

Policy makers can use the tools presented in this paper to avoid the adoption of simplistic solutions that focus only on the short term and risk increasing supply in areas that are already over-supplied. The inclusion of measures of efficiency and resource allocation could further help finding specific solutions to reduce waiting times and tackle variation. The reduction of unwarranted geographical variation in treatment rates is particularly important since variation is responsible for the allocation of resources without responding to real patient needs. It also has a great impact not only on consensus building but also on the financial sustainability of healthcare systems.

## Additional files

Additional file 1: Use rates geographical variation maps and waiting times – use rates matrixes for all the 9 ES procedures. The data includes, procedure by procedure, the geographical maps that show, by the use of colors associated to each quintile, the extent of variation in the use rates. Moreover, for each procedure, the matrix that cross-checks the waiting times and the use rates is provided. (DOCX 3008 kb)

Additional file 2: The file includes a detailed overview of elective surgery provision in Tuscany, as well as a more detailed interpretation of the findings. (DOCX 644 kb)

## Data Availability

The data that support the findings of this study are available from Tuscany regional administration but restrictions apply to the availability of these data, which were used under license for the current study, and so are not publicly available. Data are however available from the authors upon reasonable request and with permission of Tuscany regional administration.

## Abbreviations

ES: Elective Surgery
GDP: Gross Domestic Product
LHA: Local Health Authority
NHS: National Healthcare System
OECD: Organisation for Economic Co-operation and Development
PREMs: Patient Reported Experience Measures
PROMs: Patient Reported Outcome Measures
