# Supplementary figures and images for: Decision making tools for managing waiting times and treatment rates in elective surgery
Source: BMC Health Serv Res. 2019 Jun 11;19:369. doi: 10.1186/s12913-019-4199-6 (PMC6560774; doi:10.1186/s12913-019-4199-6)

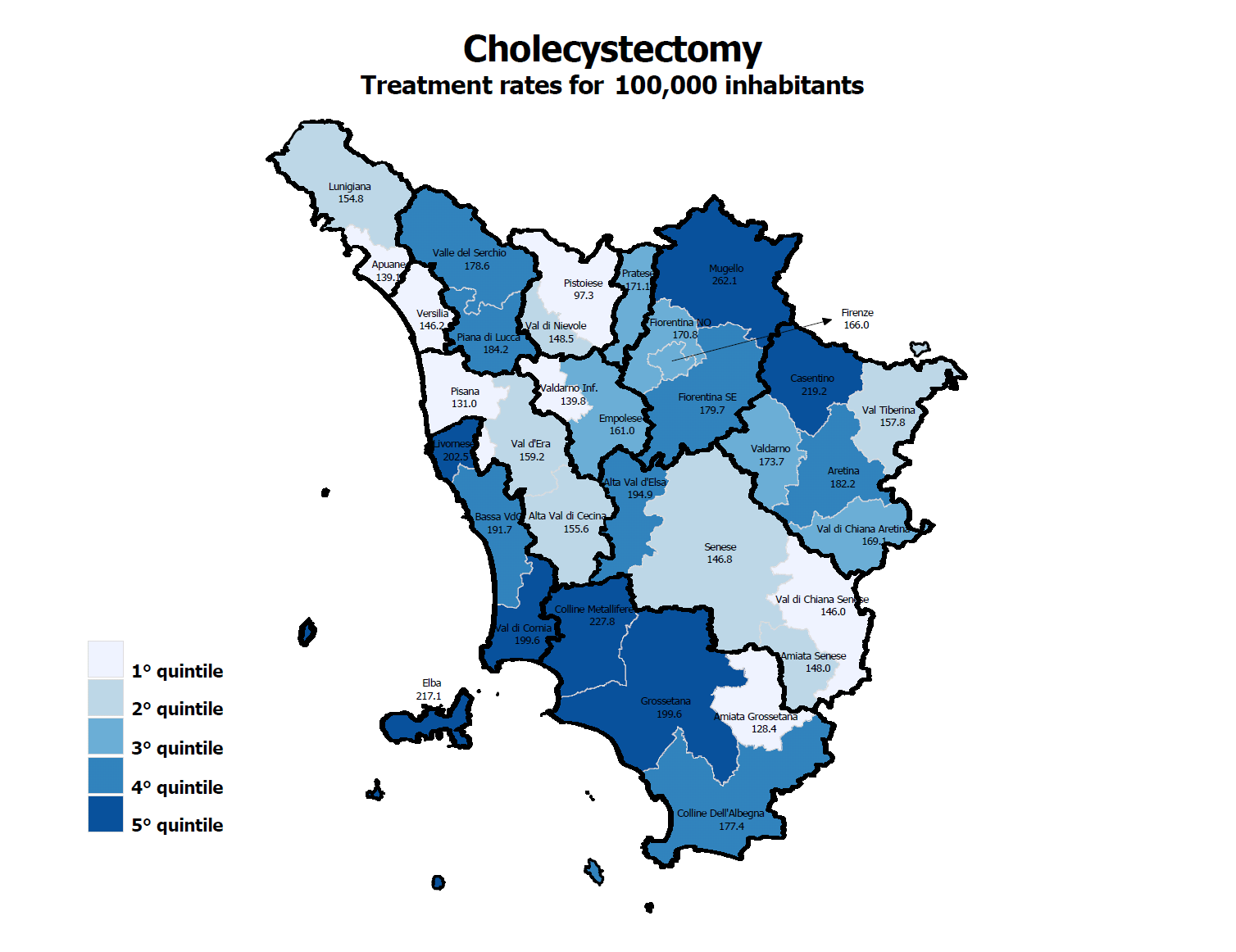


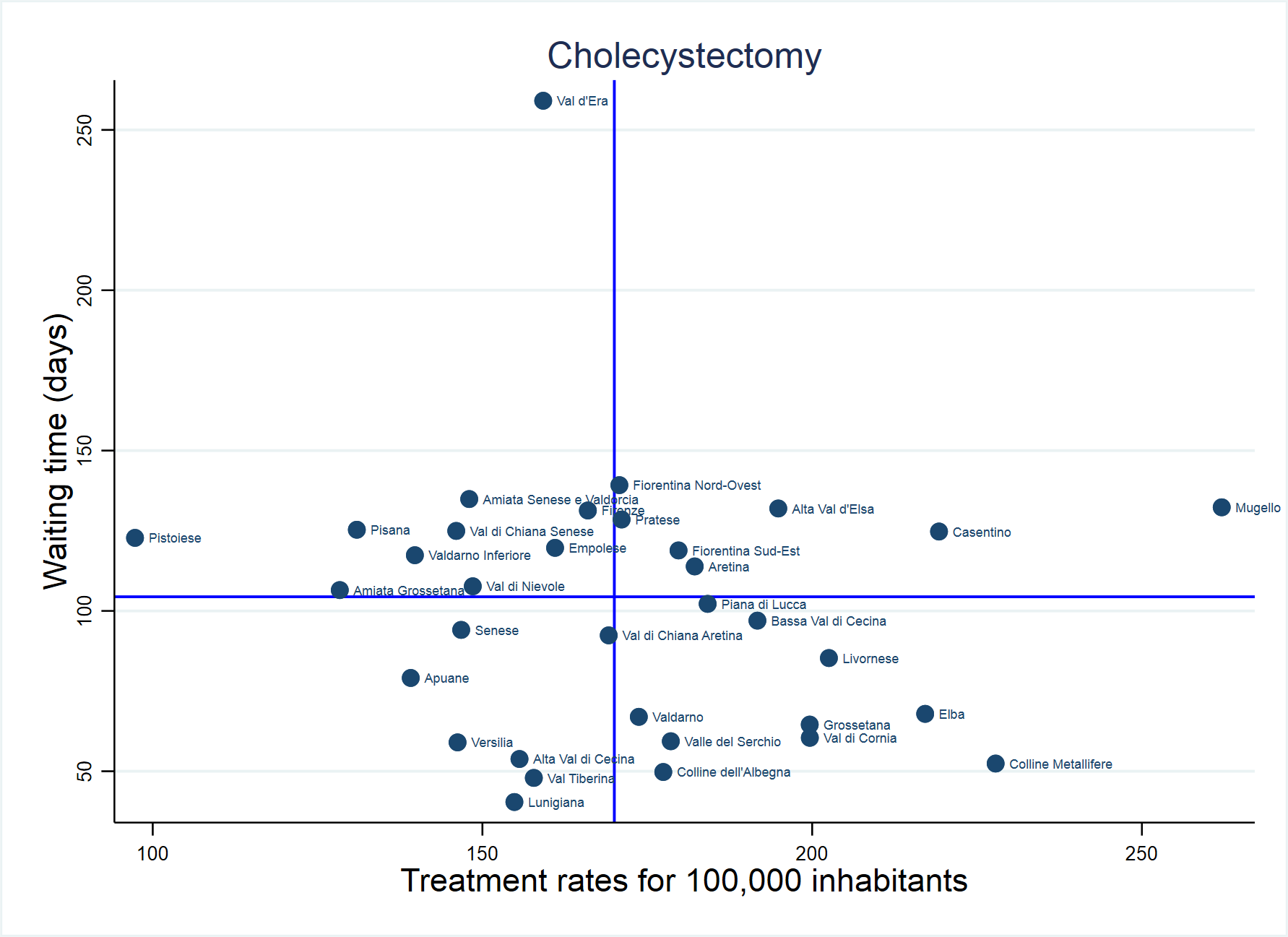


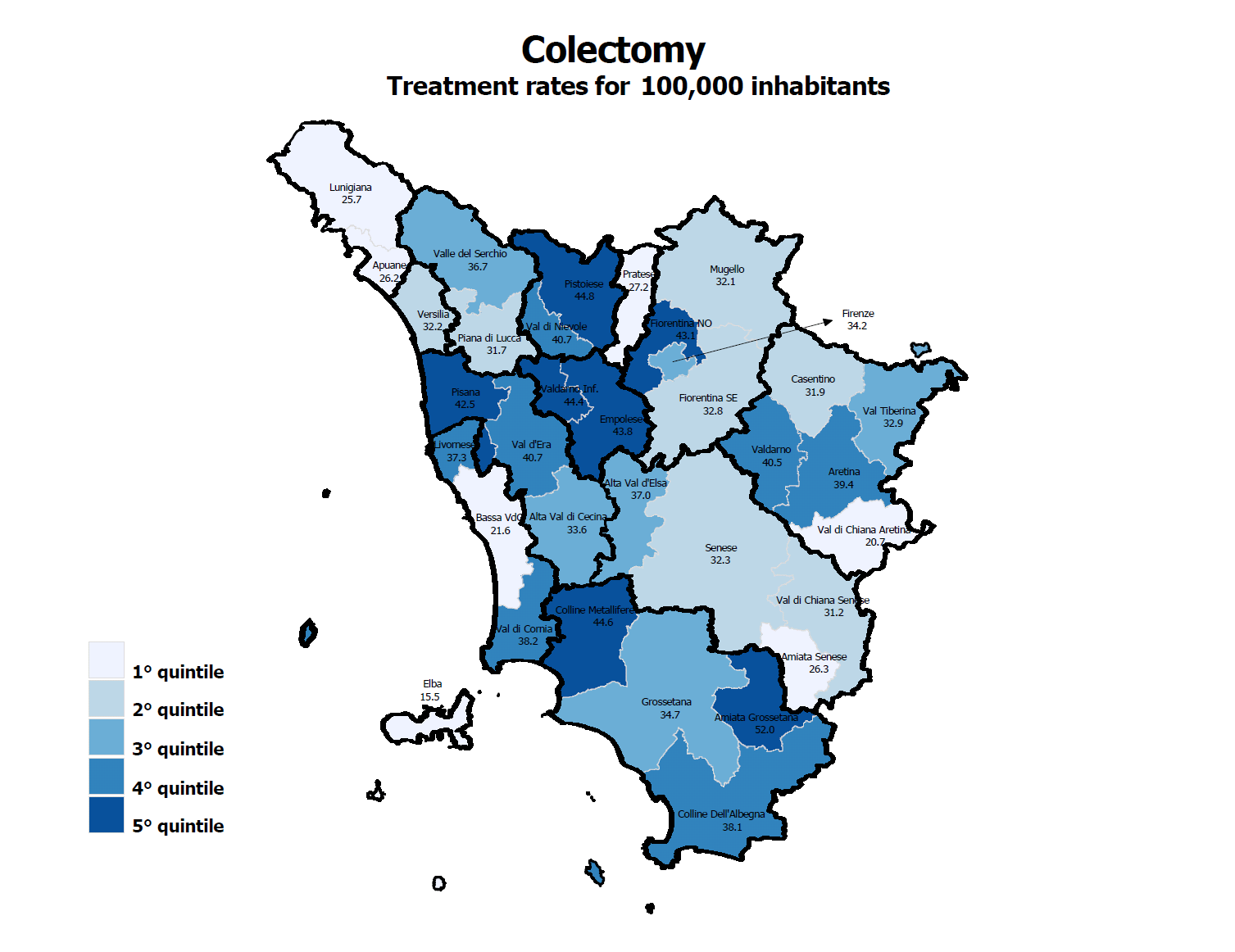


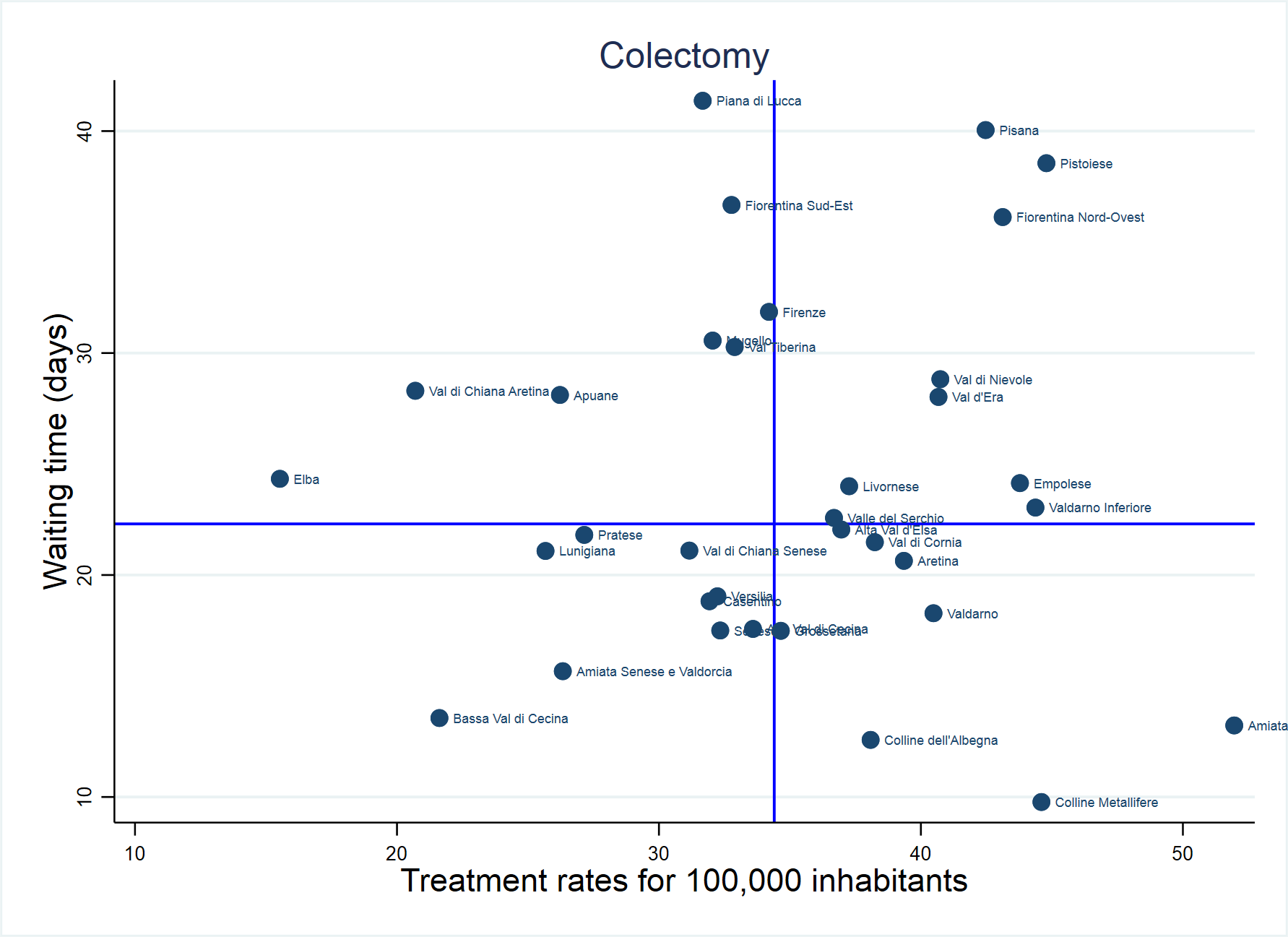


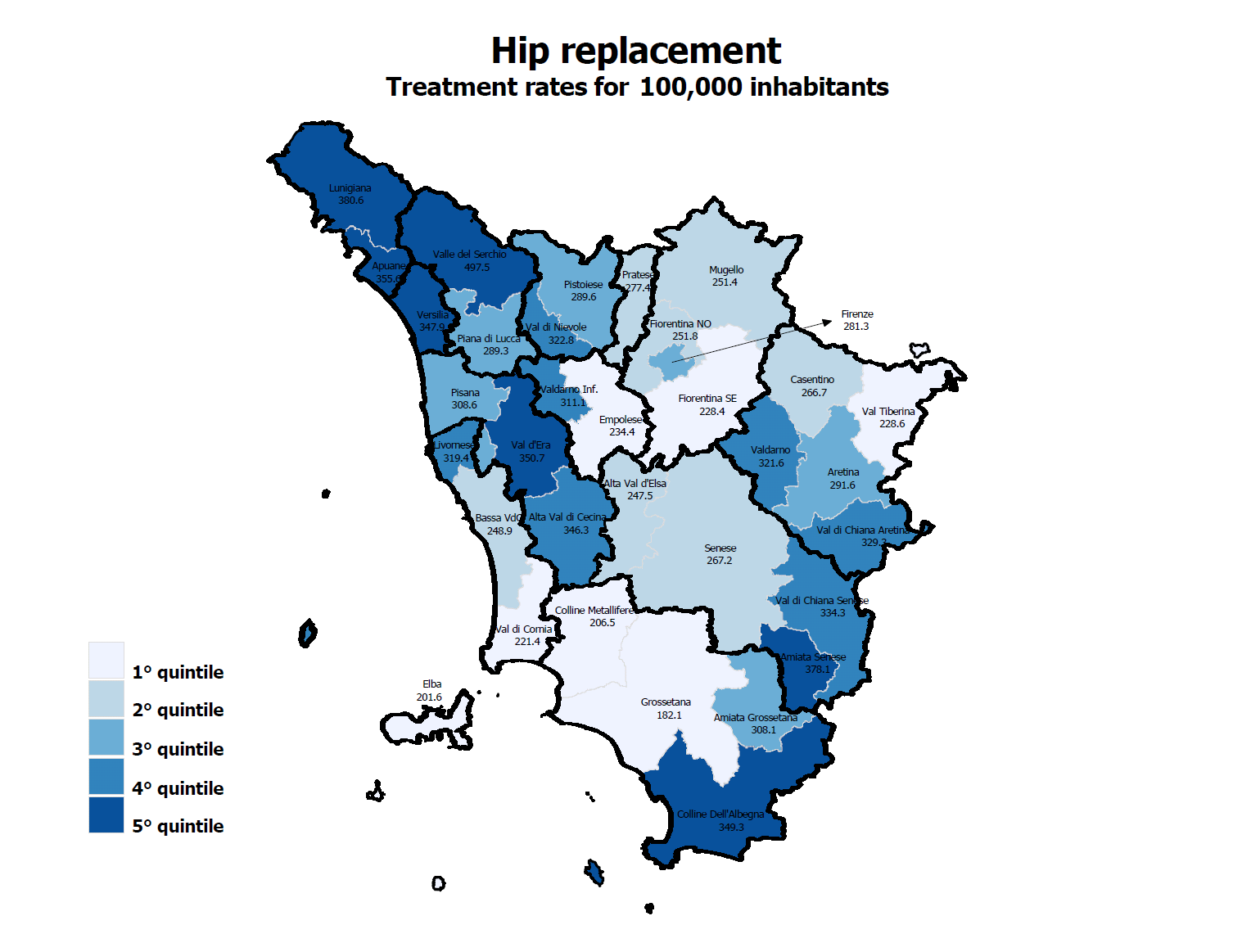


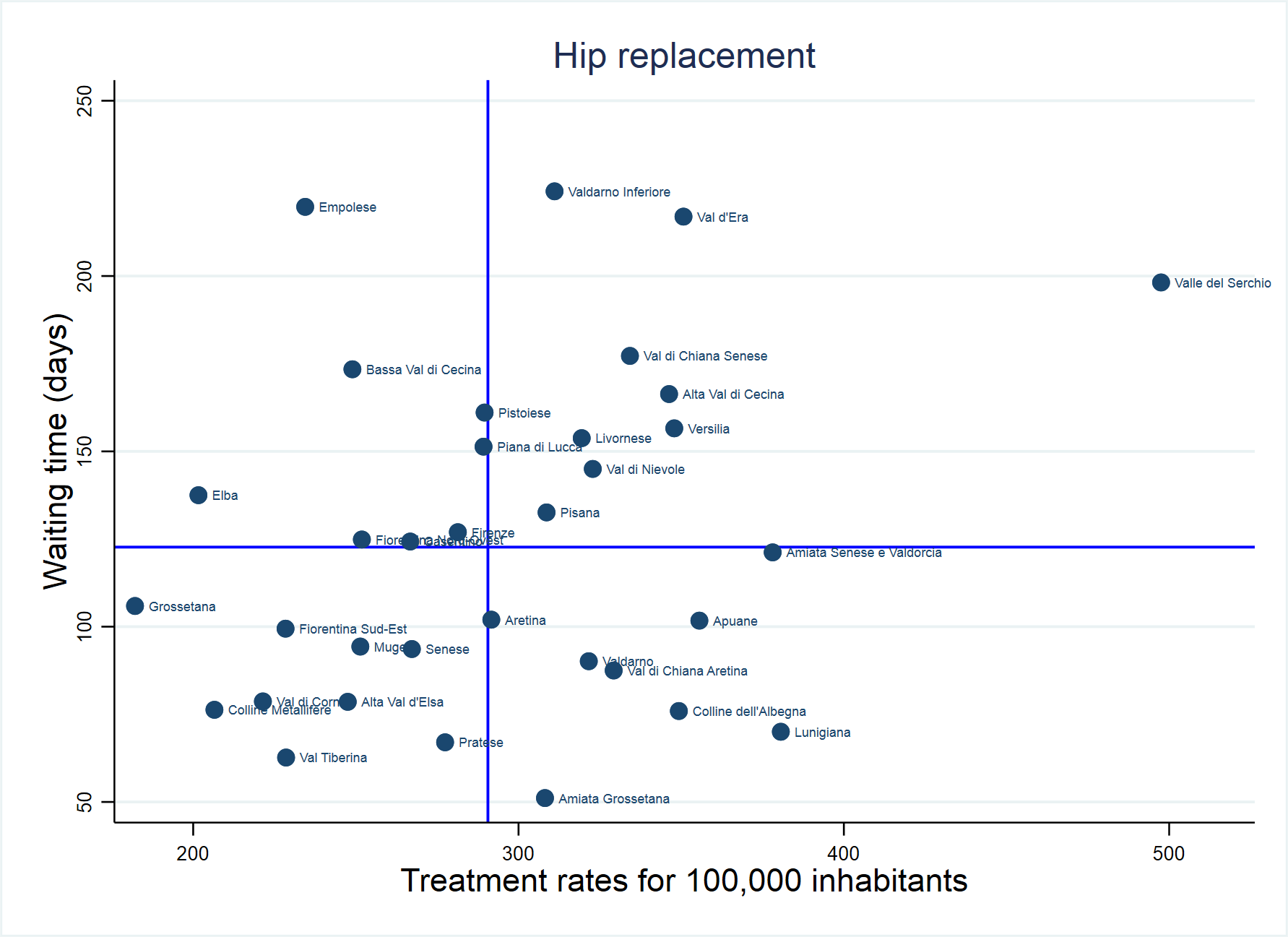


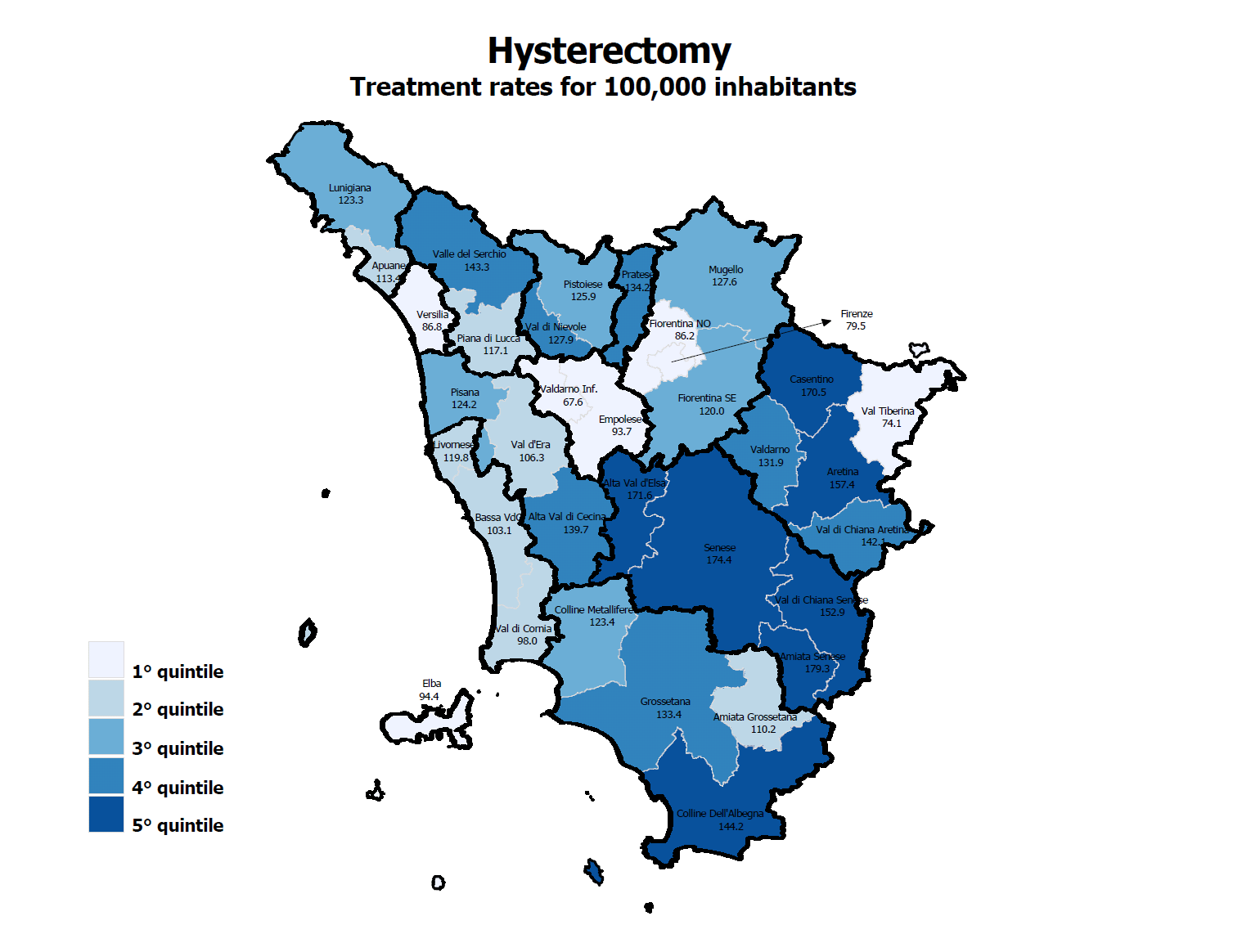


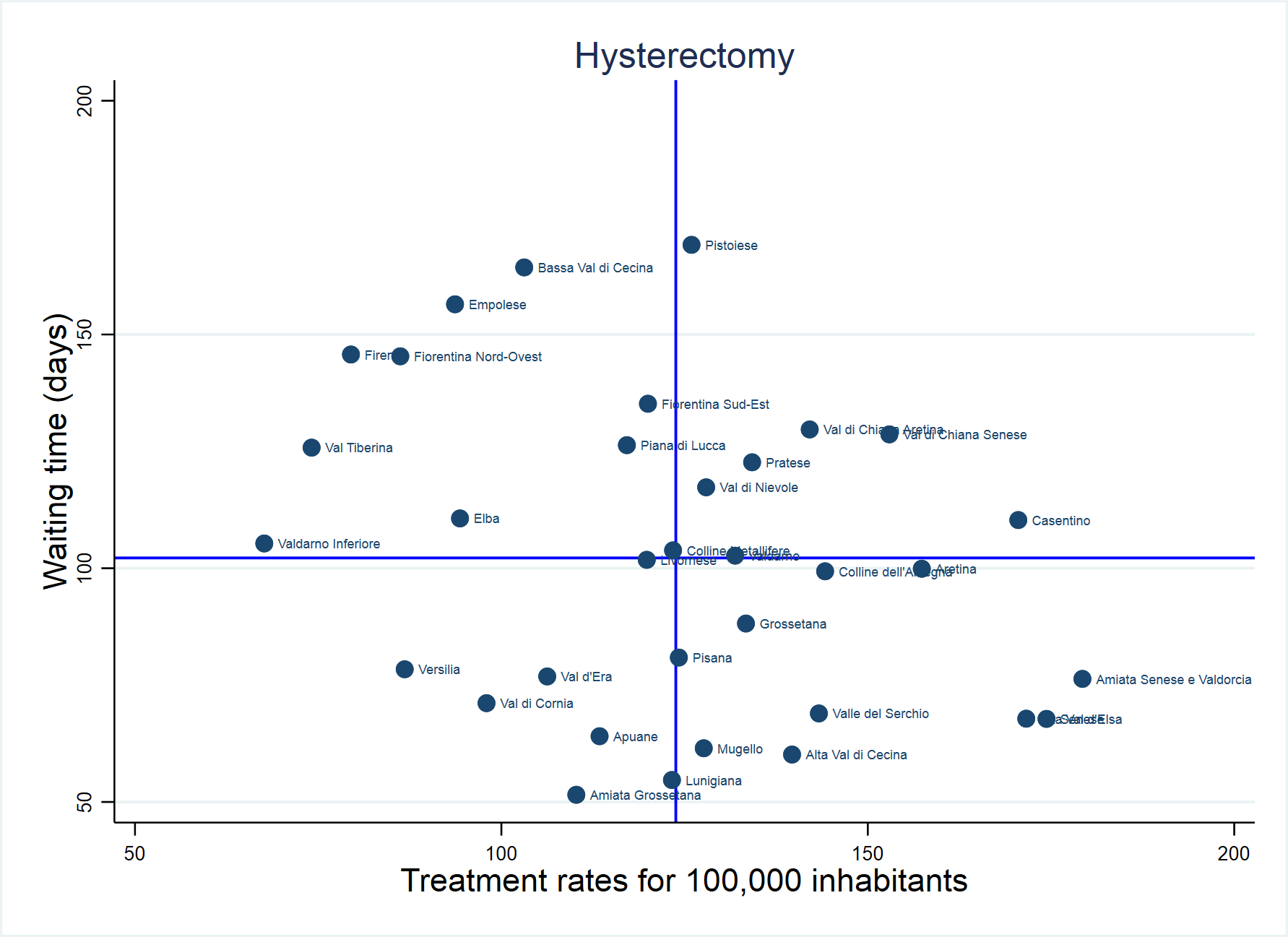


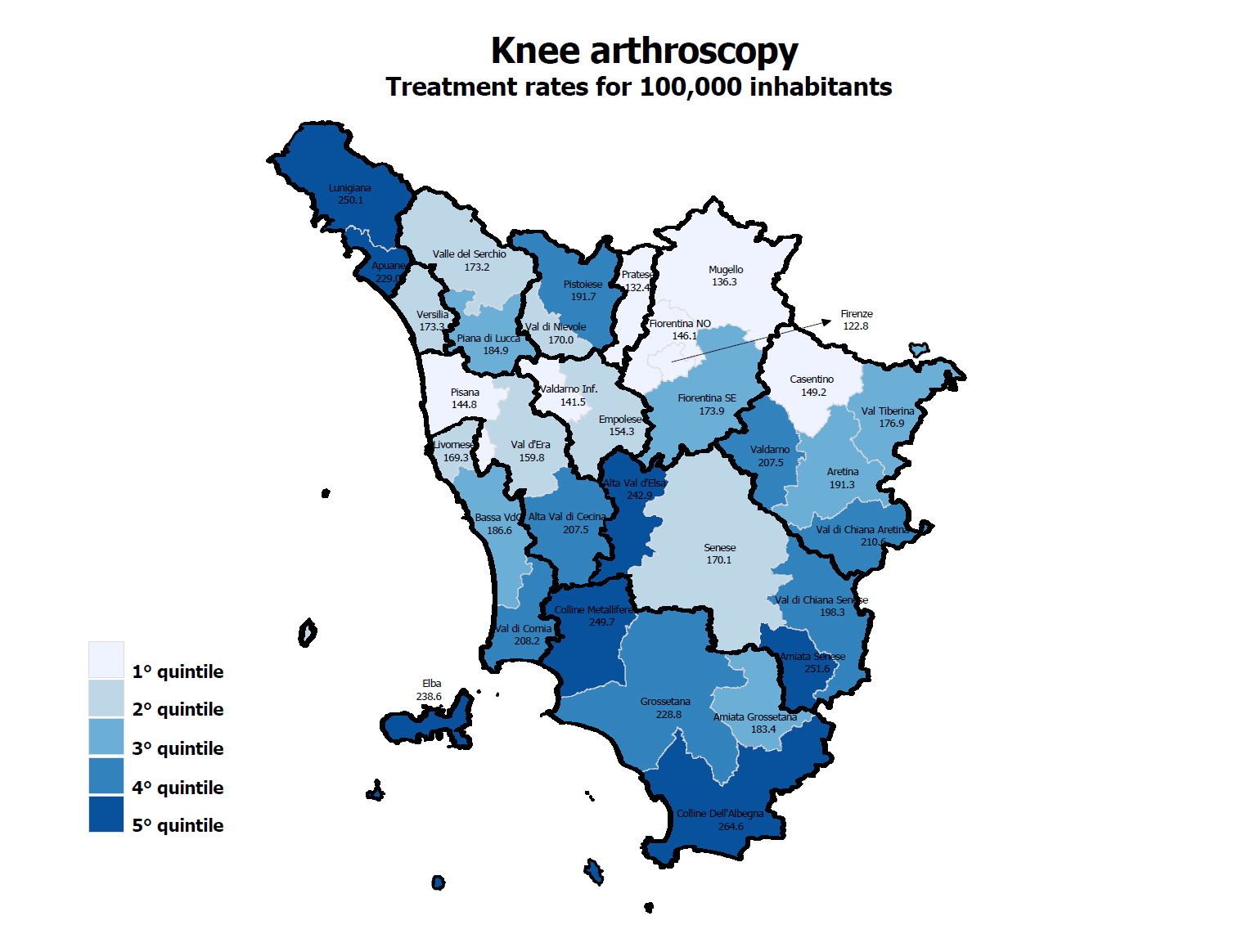

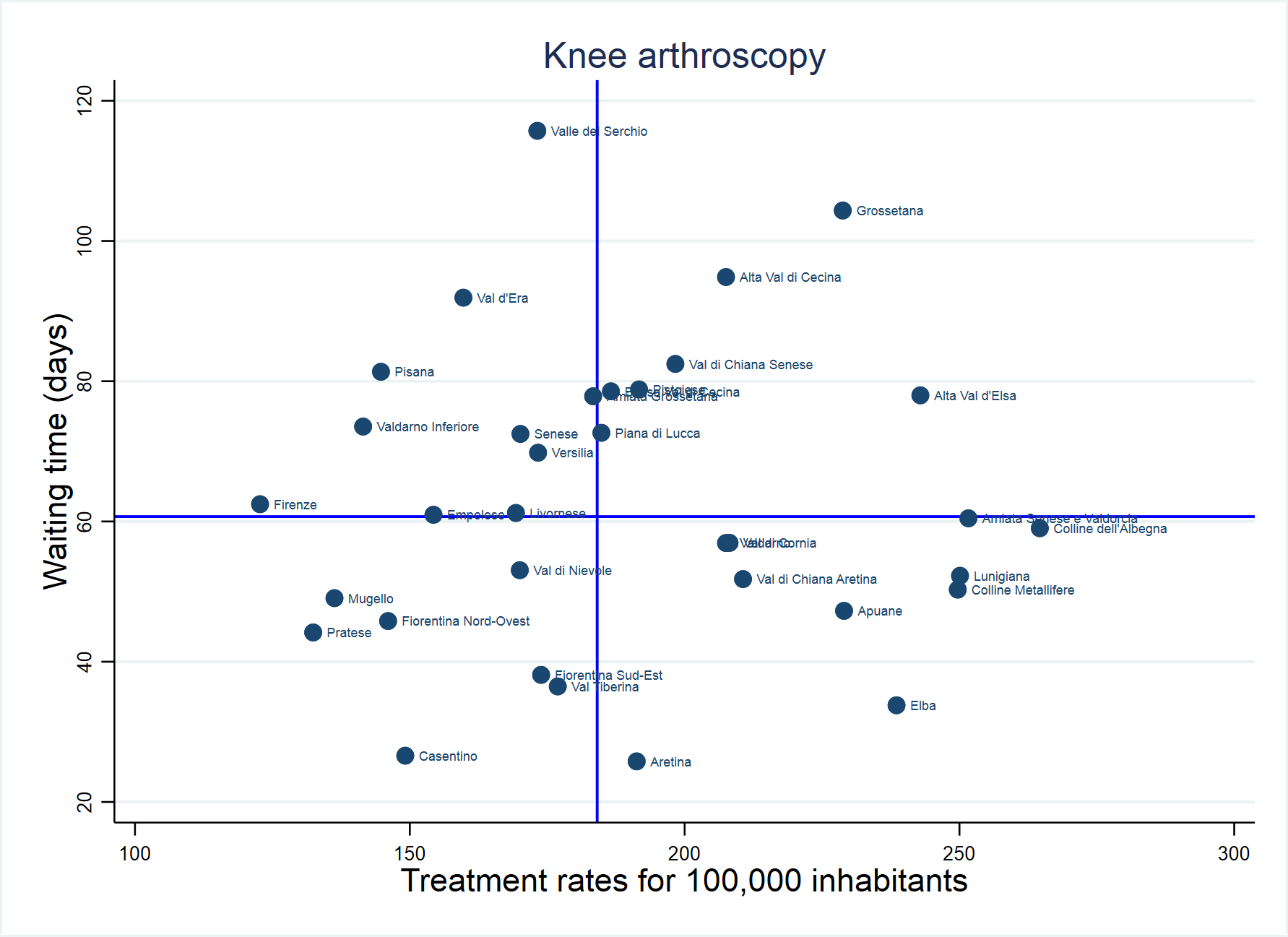


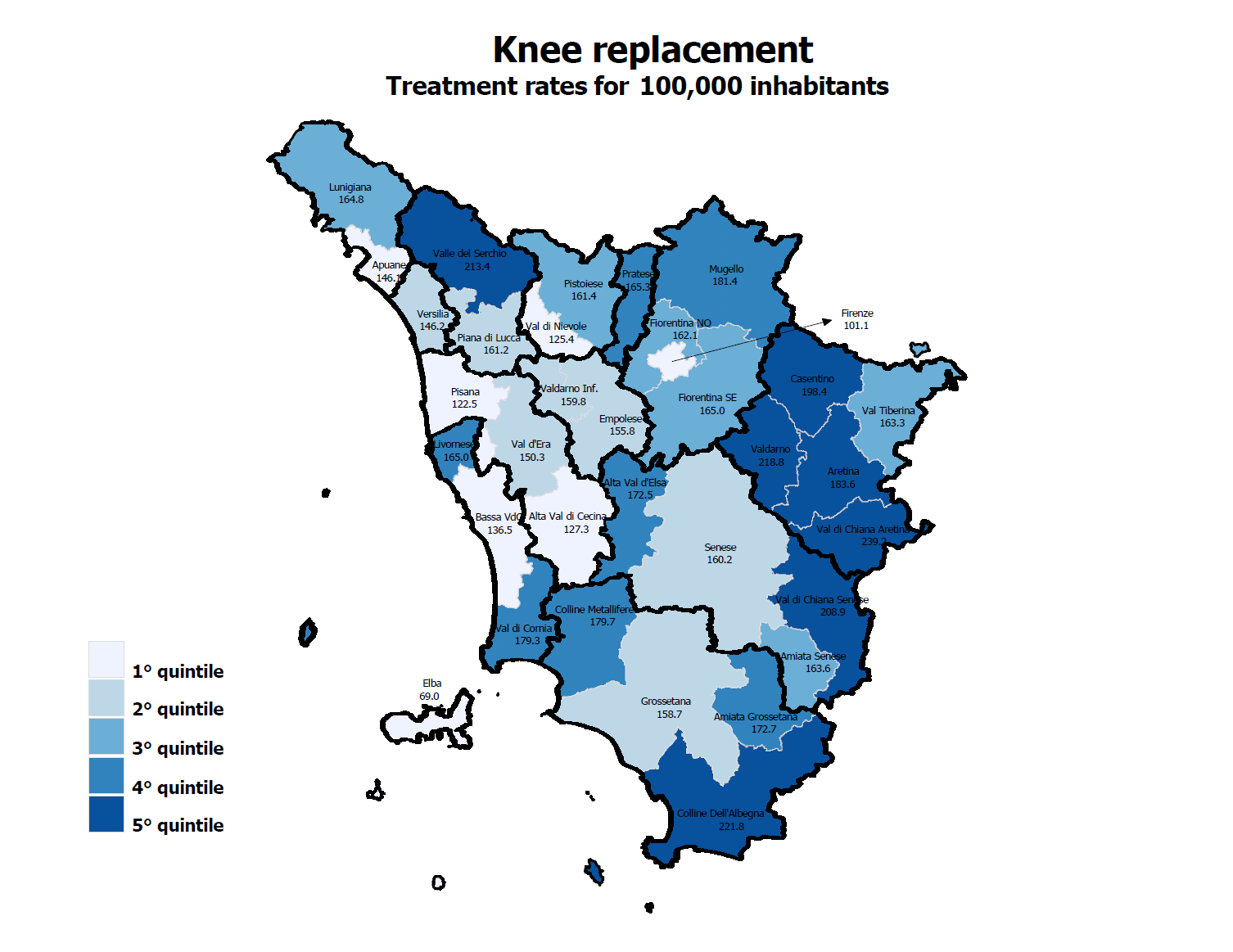

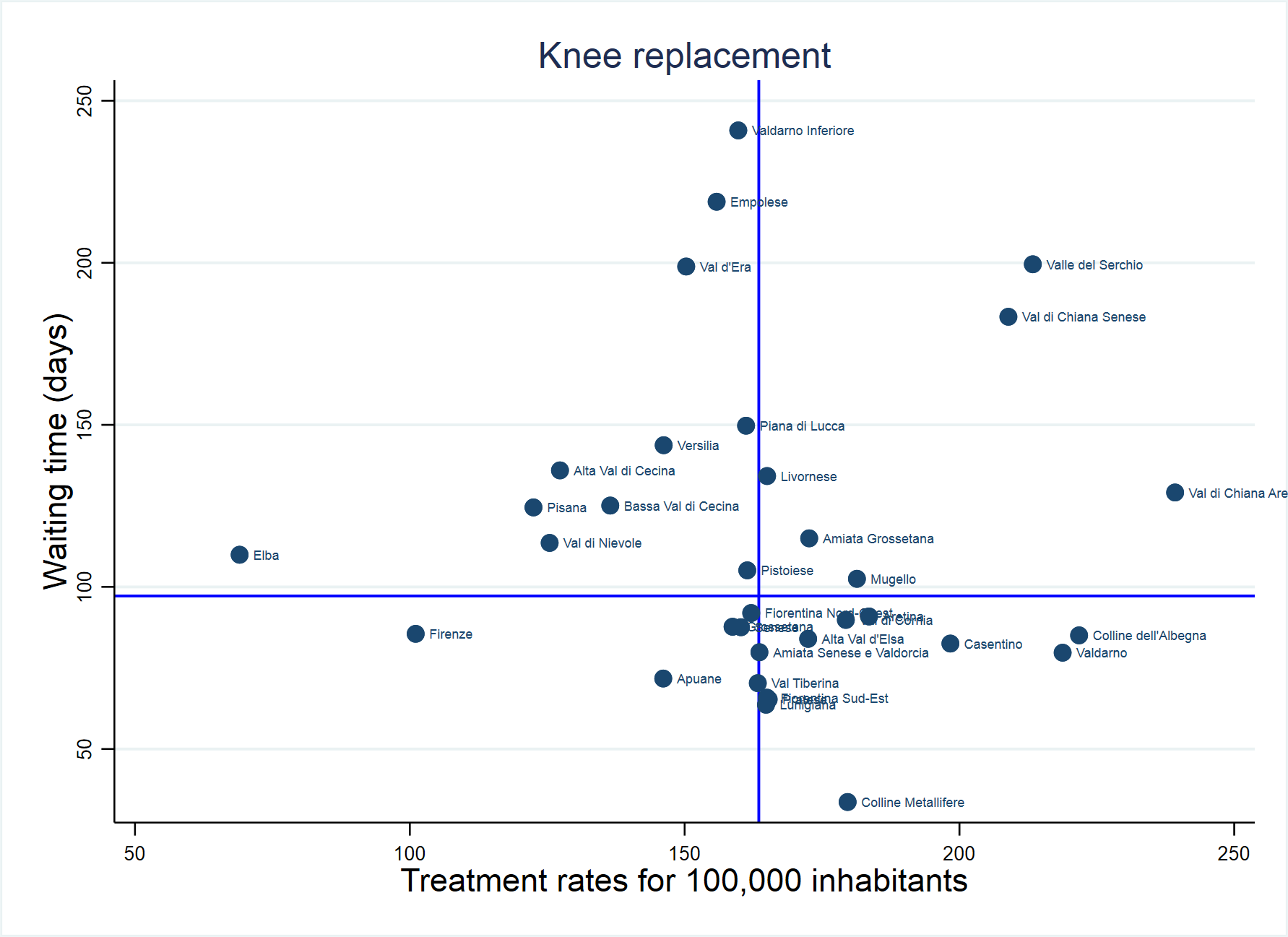


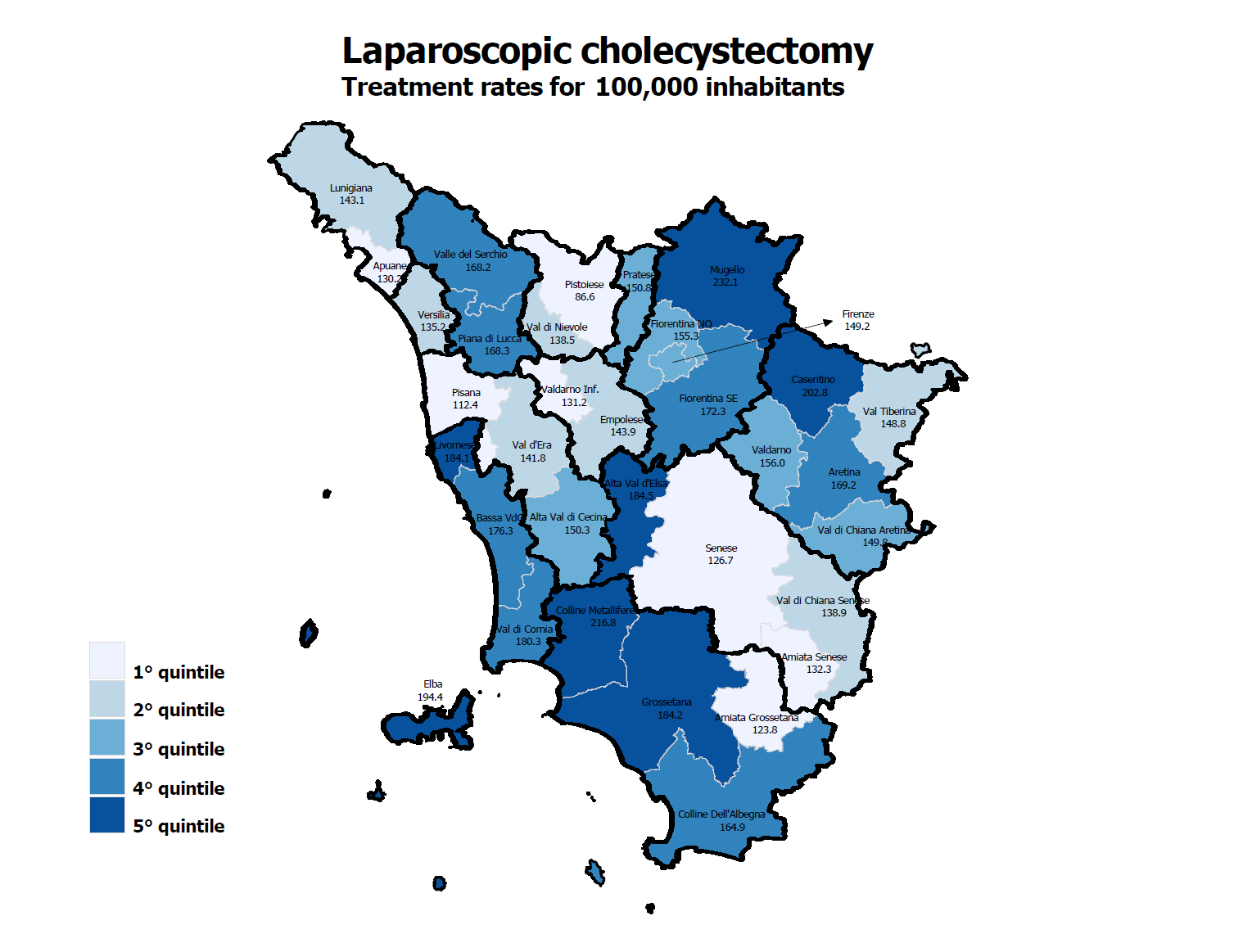


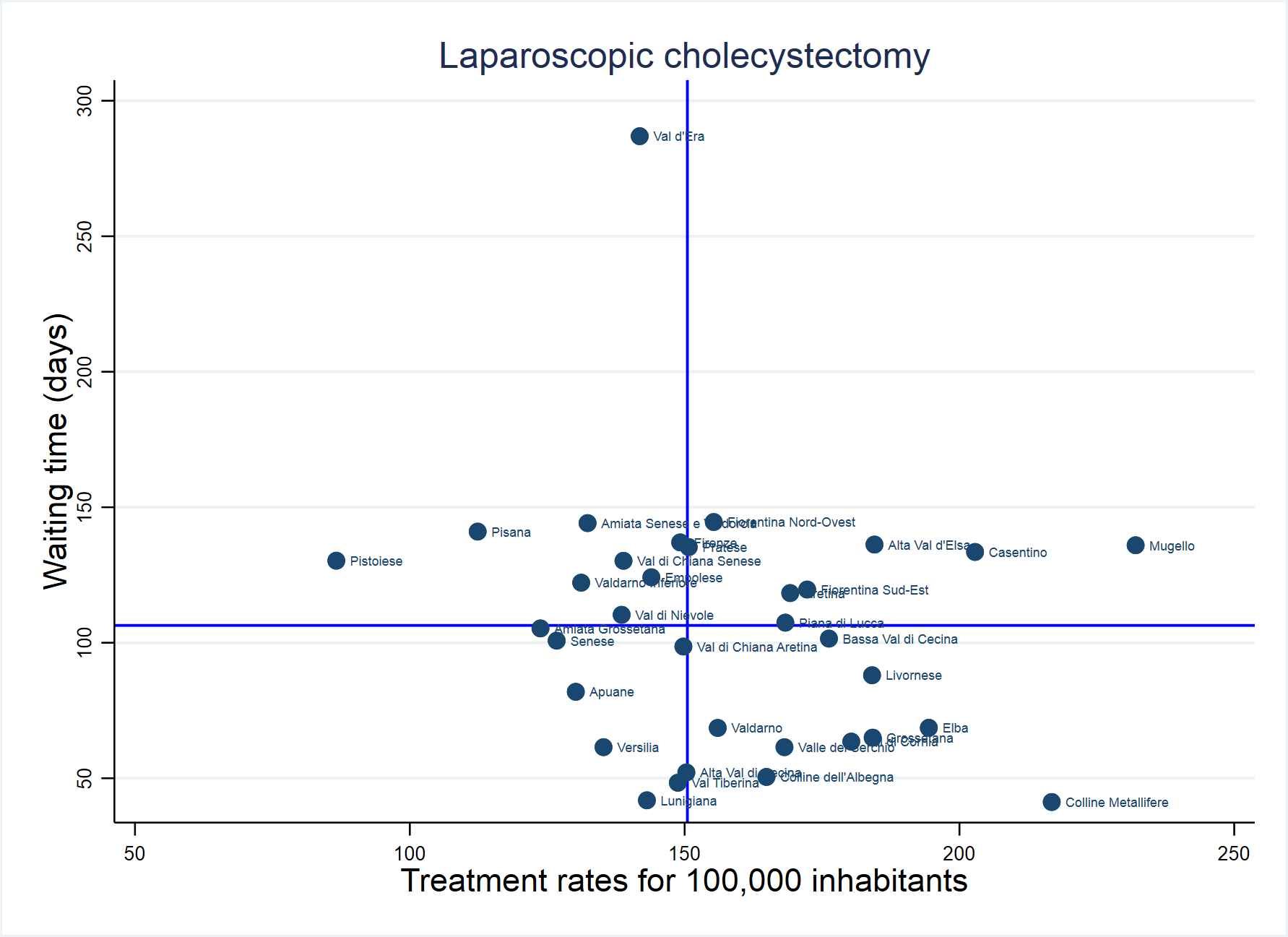


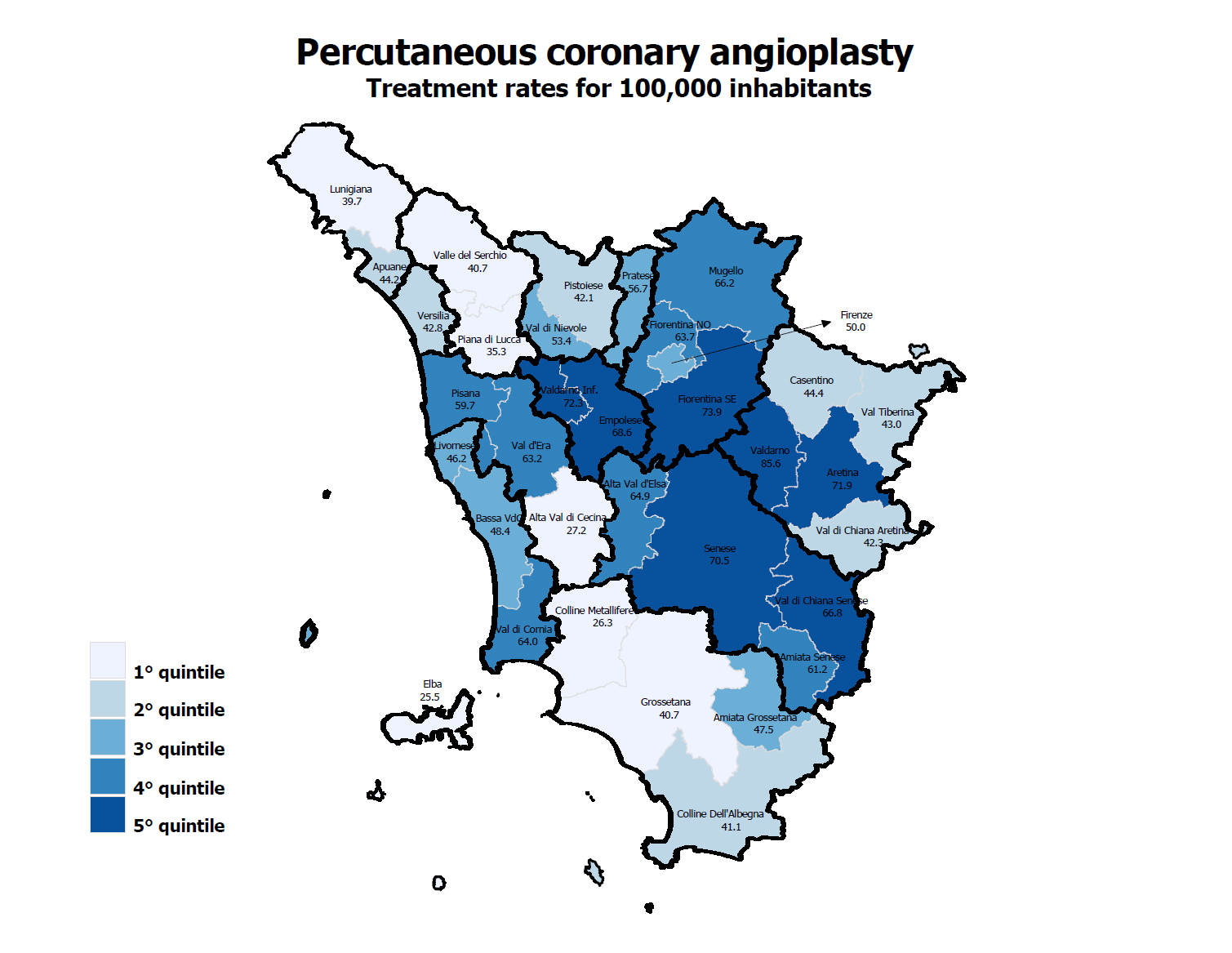

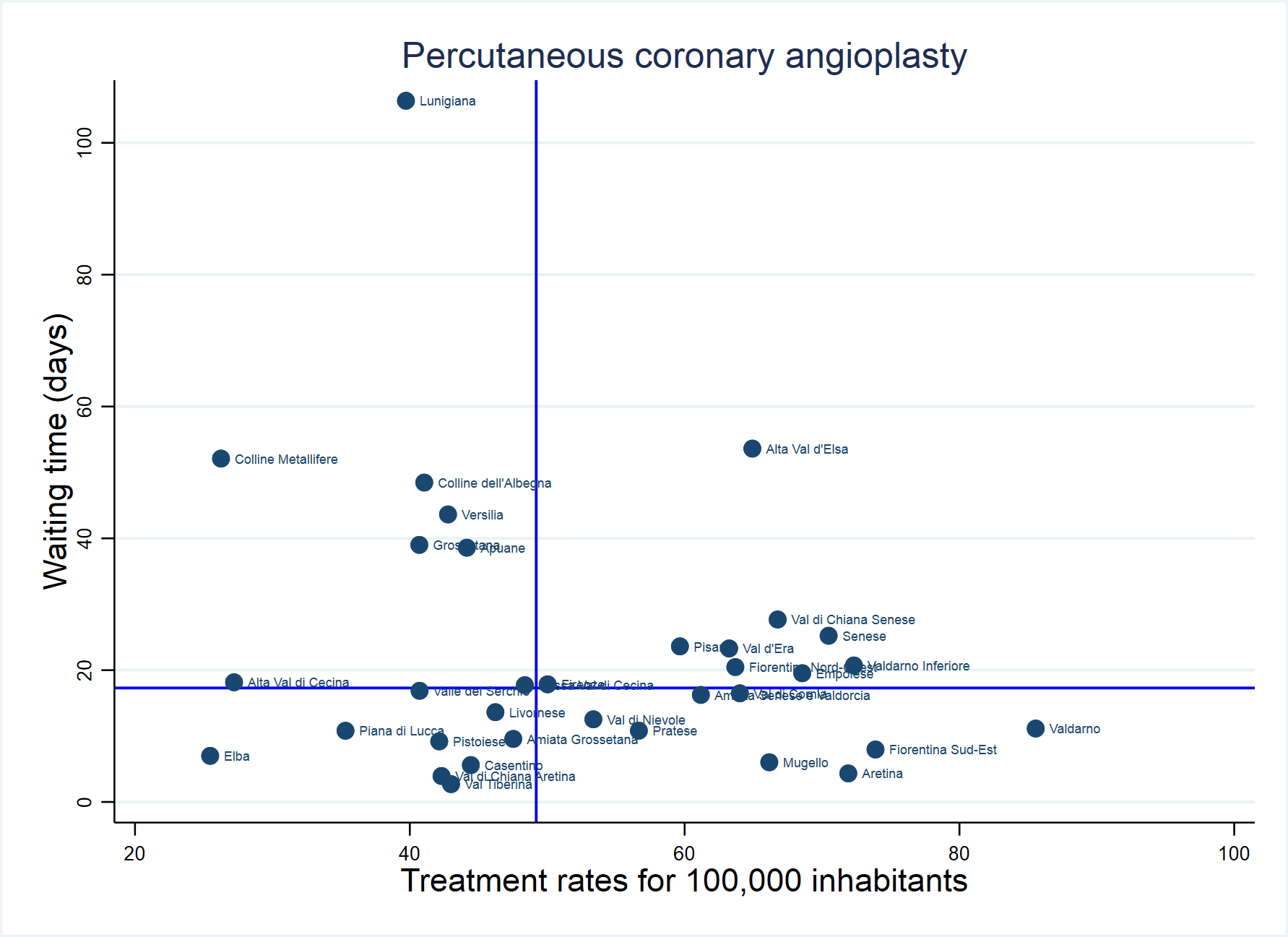


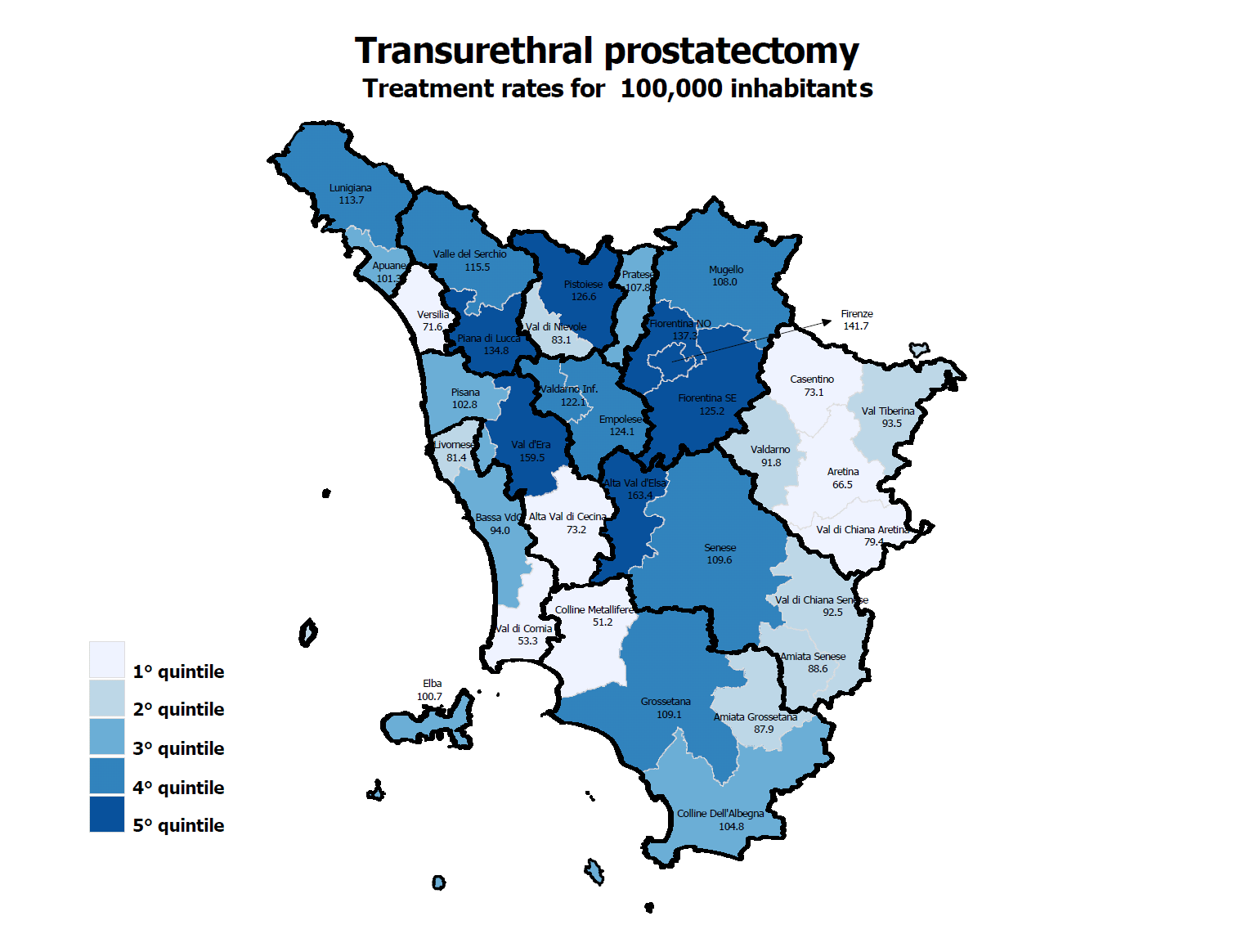


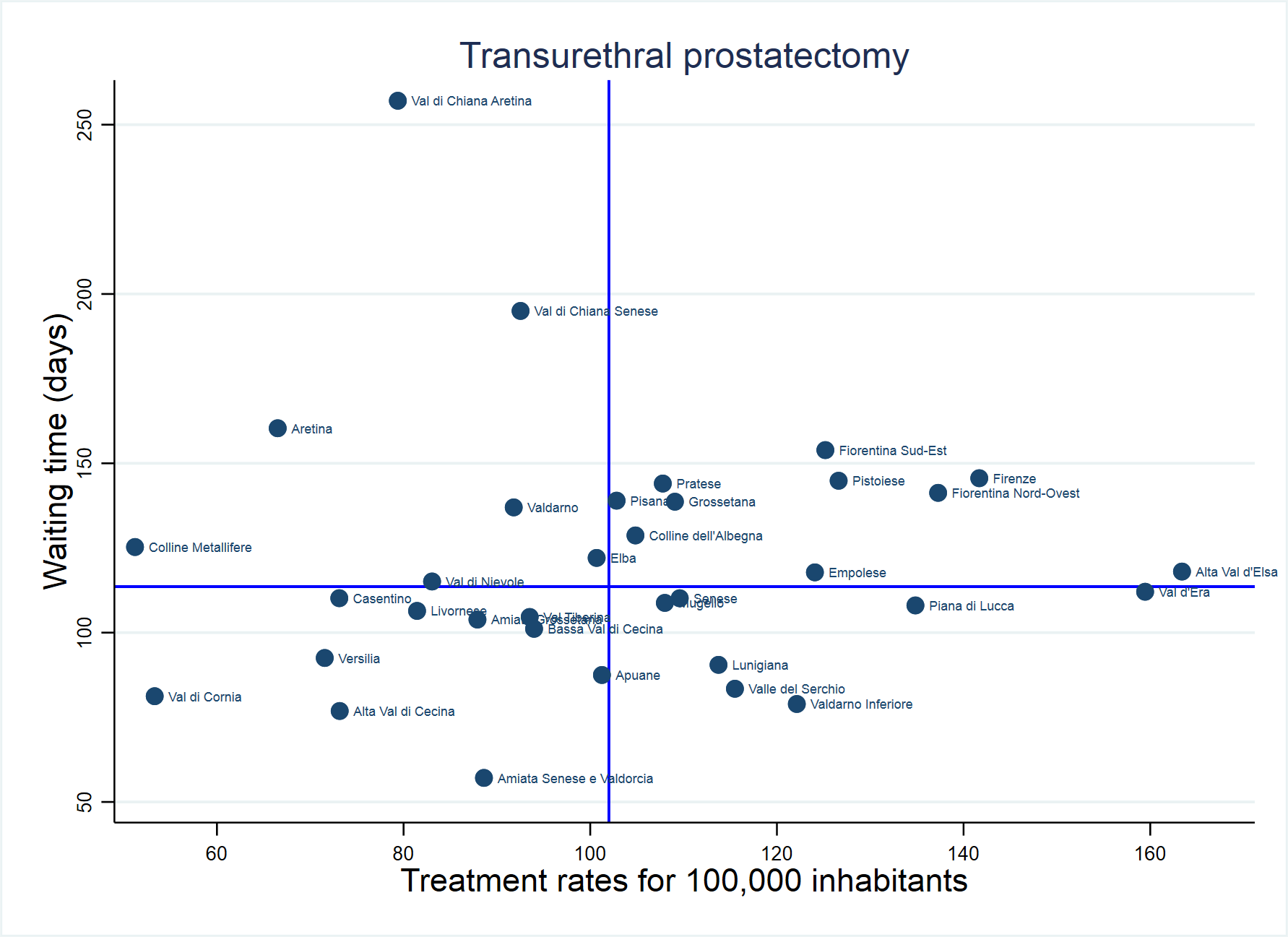

Supplement: Supplementary file 1 — Use rates geographical variation maps and waiting times – use rates matrixes for all the 9 ES procedures. The data includes, procedure by procedure, the geographical maps that show, by the use of colors associated to each quintile, the extent of variation in the use rates. Moreover, for each procedure, the matrix that cross-checks the waiting times and the use rates is provided. (DOCX 3008 kb) [file 12913_2019_4199_MOESM1_ESM.docx]
