# Supplementary material for: Decision making tools for managing waiting times and treatment rates in elective surgery
Source: BMC Health Serv Res. 2019 Jun 11;19:369. doi: 10.1186/s12913-019-4199-6 (PMC6560774; doi:10.1186/s12913-019-4199-6)
Supplement: Supplementary file 2 — The file includes a detailed overview of elective surgery provision in Tuscany, as well as a more detailed interpretation of the findings. (DOCX 644 kb) [file 12913_2019_4199_MOESM2_ESM.docx]

**ES provision and variation in Tuscany**

The Italian NHS follows a Beveridge model, based on the principle of universal coverage and financed mainly by general taxation. The resources are collected at a national level and then allocated to the 20 regions (Tuscany is a mid-size region and has 3.7M inhabitants) on a *per capita* basis, adjusted by corrective factors, such as population age. Since the 1990s, the responsibility for the organisation and provision of care has been decentralised at a regional level. The overall healthcare expenditure is 9% of the GDP, with a share of public expenditure of 75% [29].

Regions allocate resources through a capitation formula to Local Health Authorities (LHAs), which are responsible for delivering all the healthcare services (primary, home, community, and hospital care) in their geographical areas directly through their public providers or through accredited private providers. In Tuscany, there are three LHAs, which are organised into 34 health districts. Hospital care is provided by LHA-led public hospitals, private accredited hospitals and by the four university hospitals, which are autonomous bodies from the LHA where they are located. In 2016, private hospitals provided only 10% of the impatient care.

In 2016, the hospitals in Tuscany provided approximately 238,000 hospital admissions for surgical services (not considering surgical services for the Major Diagnostic Category No. 14 “Pregnancy, Childbirth and Puerperium”), of which around 173,000 (72.6%) were elective interventions and 65,000 (27.4%) were day cases.

Considering nine high-volume elective procedures (knee replacement, hip replacement, percutaneous coronary angioplasty, hysterectomy, cholecystectomy, colectomy, transurethral prostatectomy, laparoscopic cholecystectomy and knee arthroscopy), the data at the regional level hides a large variation among districts. In 2016, the average regional waiting time was 66 days, while the average waiting times at the district level ranged from 40 to 140 days. Considering a single procedure such as the knee replacement, waiting times ranged from 33 to 240 days within the 34 districts.

There is geographical variation among the 34 districts also in the treatment rates of ES services. For the nine surgical procedures mentioned above, the variation among districts in the 2016 age and gender standardised hospital admission rates ranged from two to four-fold. For instance, hysterectomy treatment rates ranged from 67.6 to 179.3 per 100,000 inhabitants (Figure 3).

Such a great variation in terms of ES standardised treatment rates has been reported persistently over time among the Tuscan geographical areas, which are homogeneous from the epidemiological and sociodemographic viewpoint (i.e. the 34 districts).

Tuscany therefore represents an appropriate context to investigate the relationship between waiting times and treatment rates in order to design specific tools to support policy makers in reducing waiting times and geographical variation.

**Hysterectomy**

Treatment rates for 100,000 inhabitants


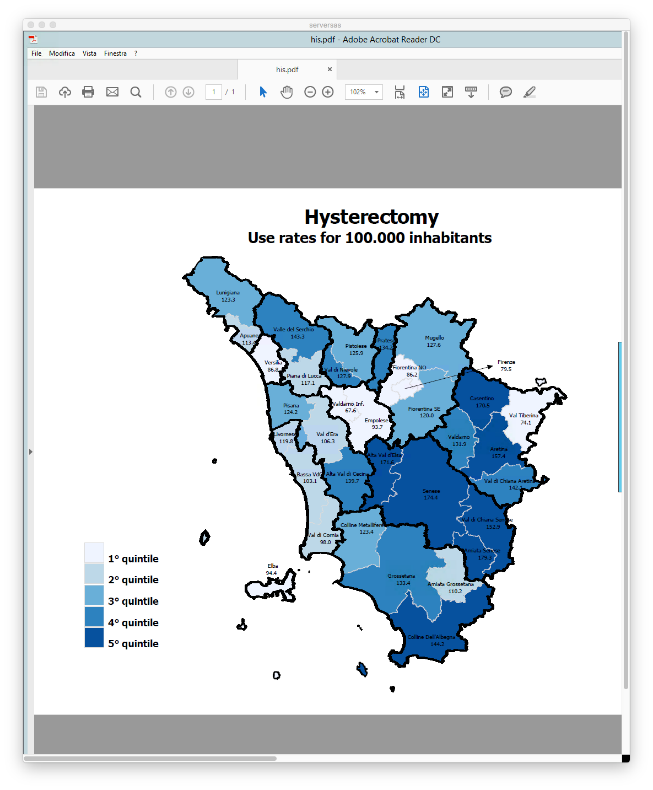


Figure 3 – An example of the variation for hysterectomy (age and gender standardised rates per 100,000 inhabitants)

Below are the overall 2016 regional data for the nine procedures delivered to Tuscan inhabitants (Table 4).

| **Procedure** | **Number of procedures** | **Average waiting time** | **Min - Max** | **Std Dev** | **Average treatment rate** | **Min - Max** | **Std Dev** |
| --- | --- | --- | --- | --- | --- | --- | --- |
| **Knee replacement** | 5173 | **112.9** | 33.6 - 240.9 | 48.1 | **162.5** | 69.0 - 239.2 | 34 |
| **Hip replacement** | 2639 | **130.9** | 51.1 - 224.2 | 47.8 | **299.7** | 182.1 - 497.5 | 63.6 |
| **Percutaneous coronary angioplasty** | 1917 | **20.1** | 2.7 - 106.4 | 20.4 | **57.7** | 25.5 - 85.6 | 15.1 |
| **Hysterectomy** | 1969 | **109.6** | 51.6 - 169.2 | 33.1 | **123.3** | 67.6 - 179.3 | 29 |
| **Cholecystectomy** | 5470 | **108.6** | 40.4 - 259.1 | 41.9 | **171.8** | 97.3 - 262.1 | 32.4 |
| **Colectomy** | 1278 | **27.4** | 9.8 - 41.4 | 8.1 | **36.8** | 15.5 - 52 | 7.9 |
| **Transurethral prostatectomy** | 1816 | **127.3** | 57.1 - 257.1 | 37.1 | **115.9** | 51.2 - 163.4 | 27.1 |
| **Laparoscopic cholecystectomy** | 4952 | **113.2** | 41.2 - 286.9 | 46.8 | **156.7** | 86.6 - 232.1 | 29.9 |
| **Knee arthroscopy** | 5264 | **62.7** | 25.8 - 115.7 | 21.3 | **180.9** | 122.8 - 264.6 | 38.7 |

Table 4 - Main figures for the nine surgical procedures provided for Tuscan inhabitants in 2016

**INTERPRETATION**

Long waiting times for healthcare services represent a source of public concern in many countries. This is true also in Italy, where the organisation and the provision of care is responsibility of each of the 20 regions. We focused on one (Tuscany and) analysed data for the 34 healthcare districts (i.e. geographical areas within the three LHAs).

We analysed nine high-volume elective surgical procedures in terms of waiting times and age and gender-standardised treatment rates and found considerable variations between the 34 districts, for both indicators.

The first finding is that waiting times and treatment rates are not correlated, suggesting that the treatment rates for ES services do not depend on waiting times. This is in line with the results reported by the international literature regarding Italy [36] and England [37].

In the healthcare sector supply can drive demand [12,13] and, therefore, different treatment rates might not be proxies for patient needs but they might be also driven by resource availability or by other factors.

To clarify the effect of supply on demand, we can differentiate between two components of the supply:

1. the productive capacity, which is given by the availability of resources (e.g. operating room hours, human resources, surgical tools);

2. the productivity level, which can be measured in terms of the volume of patients operated with respect to the available resources, which depends on the efficiency of resources [16].

In exploring the determinants of geographical unwarranted variation for ES services, the prescribing behaviour of physicians is particularly relevant as an appropriateness measure, since physicians have the power and the duty to mediate the demand for ES procedures and to manage the asymmetry in the information with the patients [38].

In this sense, the ES treatment rate (and its geographical variation) cannot be interpreted solely as an indicator of the patients’ needs, but may depend on the availability of resources, the productivity level and the extent to which physicians prescribe the intervention.

Therefore, low rates do not necessarily represent a limited demand for ES but, conversely, it may indicate inadequate patient access due to constrained resource availability, low productivity level and/or the physicians’ behaviour who are not keen to prescribe the intervention. Similarly, high rates may be the result of excessive available resources together with an extensive propensity to prescribe, instead of higher patient needs.

In order to better understand the possible strategies aimed at tackling geographical unwarranted variations, resources need to be distinguished between physical assets (operating rooms, surgical tools, diagnostic devices, etc.) and human resources (physicians, nurses, etc.). While people can be transferred from one facility to another within the same LHA, the same does not hold true in the case of most of the physical assets.

Purely supply-driven solutions aimed at increasing resource availability may be ineffective. Although strategies focusing on capacity and efficiency improvement have been widely put in place, waiting times still prevail, as do their geographical variations, and healthcare policy makers still strive to systemically shorten them. In order to reduce waiting times, policy makers and managers need to focus not only on the internal efficiency of individual providers but also on the geographical variation in patient access. Another key element that has to be taken into consideration is the physicians’ engagement as they have a central role in the improvement process.

The interrelation between the geographical variation in treatment rates and in waiting times thus enabled us to identify different scenarios where waiting times should be tackled through different strategies.

To explore the interrelation between treatment rate and waiting times, we applied the framework proposed by Nuti and Vainieri (2012) to elective surgical procedures, by plotting the performance of each health district in Tuscany in a waiting time – treatment rate matrix.

**Figure 1b -** Logical framework to help managers deal with waiting times and treatment rates

The bottom-right quadrant (No. 4) contains those districts who suffer from an overuse problem, an excessive supply structure, or both. The inhabitants of these districts receive much care and face relatively short waiting times. The combination of low waits and high rates could be tackled by a reallocation (reduction) of resources, either between the medical specialties within a district or directly between districts, without causing a substantial increase in waiting times. The reallocation, rather than the addition, of resources would lead to an improved performance without increasing costs [35]. As previously stated, while the reallocation of physical assets is generally more problematic, the reallocation of people within the same LHA is more feasible. As an example, on the basis of the organisational Tuscan Healthcare reform in January 2016, a regional law foreseeing the mobility of the medical staff within the LHA was adopted in November 2016 (Decision of the Regional Council n. 1200 of 29/11/2016). Consequently, regional policy makers are able to put in place a more efficient reallocation of human resources.

The districts positioned in the bottom-left quadrant (No. 3) have short waiting times, low treatment rates and therefore may suffer from underuse. Although people receive care timely, the treatment rates are below the regional median value and managers should therefore understand whether all the need is identified and channelled into the system. This may be due to an inadequate productivity or to an insufficient propensity of physicians to prescribe the intervention to patients. In these cases, the improvement of providers’ productivity or a shared discussion between professionals regarding the prescribing guidelines may be effective in shifting rates towards the regional standards without causing an increase in waiting times.

In the third and fourth quadrants, waiting times are relatively short and the districts should tackle the variations in treatment rates. These districts should mainly work on the different propensity of physicians to prescribe the surgical intervention, also focusing on productivity levels in the third quadrant and resources availability in the fourth one. Obtaining the physicians’ engagement in the improvement process is fundamental to disclose appropriateness results, discuss the guidelines and align their prescribing behaviour with the organisational/regional strategy.

The upper-right quadrant (No. 2) contains the districts that have high rates and long waiting times. The high rates suggest potential overtreatment, and managers should firstly investigate why so many patients are being entered into the system but also address potential resource misallocation, similarly to quadrant 1. Supply-side interventions aimed at reducing waiting times by increasing the productive capacity are not advisable. In fact, they would probably lead to either an increase in treatment rates, which would saturate the additional capacity leaving waiting times unchanged, or to an actual reduction in waiting times in those areas that probably account for an already excessive resource allocation with respect to other districts positioned in the quadrants on the left side of the matrix. Instead, strategies focusing on the prescribing behaviour of physicians could have a beneficial impact on shortening waiting times and on the appropriate use of elective surgical services.

Districts belonging to the upper-left quadrant (No. 1) are characterised by long waits and low treatment rates. Because of the long waiting times, in these districts citizens may perceive a barrier to accessing healthcare services and therefore may opt not to be treated. Even though the system is identifying need, there is a potential problem of supply constraints (or scarce productivity levels) and managers should investigate why that need ends up on the waiting list rather than being treated. A possible solution would be to strengthen the supply structure and/or to enhance productivity. This would enable districts to increase treatment rates while reducing waiting times, producing a net benefit for the citizens.

“Pure” supply-side strategies aimed at increasing the resource availability therefore only seem beneficial for those districts where long waiting lists are associated with a relatively low patient access.

Hence, by using the matrix tool, policy makers and managers can interpret results, collect additional data and then propose specific actions for each district. The matrix can thus be used to evaluate whether “pure” supply-side interventions are able to shorten waiting times and to avoid additional resource allocations that would contribute to widening the geographical variation and inequity. This is particularly important in a context where healthcare systems face tight resource constraints. The matrix also provides an effective picture of the performance of each district for a specific procedure and can help policy makers and managers to understand what scenario each district belongs to.

In addition, the summary table provides a broader picture and a systemic review of the issues highlighted in the nine matrixes. It includes the performance of each district for all the procedures included in the study. Hence, it can be used to evaluate whether districts face organisational problems for all the surgical activities or whether criticalities appear for some procedures only.

Taking into consideration the example of district number 1 shown in table 2, a resource reallocation among procedures seems appropriate (e.g. increasing the operating room hours for knee replacements while reducing the slots allocated to colectomy). This reallocation would imply an increase in the treatment rate of knee replacement and, simultaneously, a reduction of waiting times.

The reallocation of resources needs to be managed not only at a district or specialty level, but might also need to be discussed at a broader LHA or regional level. The choice of the right level depends on the organisational model of the hospital network. If most of the inhabitants use the hospitals led by their district, the reallocation can be performed at a district level, involving the management and physicians from district hospitals to review the ES planning.

If, on the other hand, a high share of inhabitants of one district move towards hospitals in different districts to receive care, then reallocation, in terms of reducing waiting times and geographical unwarranted variation, needs to be managed at a higher decision-making level than the district one. If this is the case, the ES delivery planning should bring together the resources of several districts and involve the management and physicians from different districts within a system and population-centred perspective. In Beveridge healthcare systems that stipulate the patients’ right to choose the hospital where they are treated, ES planning at a system level seems particularly relevant.

Lastly, we recommend the use of the matrix and the summary table in order to guide policy makers and managers when dealing with resource (re)allocations aimed at reducing geographical variations in waiting times and treatment rates in order to ensure timely care and improve equity at a system level.
